# Drug-Induced Electrolyte Disturbances in Older Adults Presenting to the Emergency Department: Mechanisms, Recognition and Prevention

**DOI:** 10.3390/jcm15218719

**Published:** 2026-11-08

**Authors:** Alexandru Cristian Brici, Maria Octavia Murariu, Alexia Manole, Raul Patrascu, Vera Ciocan, Dorin Novacescu, Talida Georgiana Cut, Alina Petrica, Felicia Manole, Flavia Zara, Cristina Stefania Dumitru, Marius Liviu Moise

**Affiliations:** 1 Department of Doctoral Studies, Victor Babes University of Medicine and Pharmacy Timisoara, E. Murgu Square, No. 2, 300041 Timisoara, Romania; alexandru.brici@umft.ro (A.C.B.); octavia.brici@umft.ro (M.O.M.); 2 Emergency Unit, County Emergency Hospital Resita, 320154 Resita, Romania; 3 ENT Department, County Emergency Hospital Resita, 320154 Resita, Romania; 4 Faculty of Medicine and Pharmacy, University of Oradea, 410087 Oradea, Romania; manole.alexia@student.uoradea.ro; 5 Department of Functional Sciences, Victor Babes University of Medicine and Pharmacy Timisoara, E. Murgu Square, No. 2, 300041 Timisoara, Romania; patrascu.raul@umft.ro; 6 Department VIII, Discipline of Forensic Medicine, Bioethics, Deontology and Medical Law, Victor Babes University of Medicine and Pharmacy Timisoara, E. Murgu Square, No. 2, 300041 Timisoara, Romania; veronica.ciocan@umft.ro; 7 Department II of Microscopic Morphology, Discipline of Histology, Victor Babes University of Medicine and Pharmacy Timisoara, E. Murgu Square, No. 2, 300041 Timisoara, Romania; novacescu.dorin@umft.ro (D.N.); flavia.zara@umft.ro (F.Z.); 8 Department XIII, Discipline of Infectious Diseases, Victor Babes University of Medicine and Pharmacy Timisoara, E. Murgu Square, No. 2, 300041 Timisoara, Romania; talida.cut@umft.ro; 9 Centre for Artificial Intelligence and Big Data in Emergency Medicine and Clinical Research, Department of Surgery, Victor Babes University of Medicine and Pharmacy, 300041 Timisoara, Romania; alina.petrica@umft.ro; 10 Department of Surgery, Victor Babes University of Medicine and Pharmacy, 300041 Timisoara, Romania; 11 ENT Department, Faculty of Medicine and Pharmacy, University of Oradea, 410068 Oradea, Romania; fmanole@uoradea.ro; 12 Radiology Department, Emerald Medical Center, Corneliu Coposu Boulevard 14, 300552 Timisoara, Romania; moiseliviumarius@gmail.com

**Keywords:** older adults, emergency department, adverse drug events, hyponatremia, hyperkalemia, hypomagnesemia, thiazide diuretics, polypharmacy, deprescribing, point-of-care ultrasound

## Abstract

Many older adults attending the emergency department (ED) take medications that interfere with water and electrolyte balance. This narrative review, based on a structured PubMed/MEDLINE search covering January 2016 to September 2026, summarizes evidence on drug-associated disorders of sodium, potassium, magnesium and calcium in older ED patients. Thiazides, antidepressants, antiepileptic drugs, antipsychotics, proton pump inhibitors and desmopressin are associated with hyponatremia; with thiazides, the risk peaks in the first weeks of treatment and is highest in women aged 80 years or older. Lithium can cause nephrogenic diabetes insipidus, which may persist after discontinuation and cause hypernatremia when water intake is inadequate. Renin–angiotensin–aldosterone system inhibitors, mineralocorticoid receptor antagonists, trimethoprim and non-steroidal anti-inflammatory drugs predispose to hyperkalemia, particularly in chronic kidney disease; diuretics and proton pump inhibitors are associated with hypokalemia and hypomagnesemia; and denosumab can cause severe hypocalcemia in advanced kidney disease. Most of this evidence is observational, and drugs usually act together with comorbidities, acute illness and other medications. We propose a severity-based ED algorithm integrating medication history, laboratory, electrocardiographic and volume assessment (including point-of-care ultrasound) to guide stabilization, drug adjustment, safe correction and disposition. ED-based medication safety programs reduce inappropriate prescribing, but their effect on electrolyte-related outcomes remains unproven.

## 1. Introduction

Older adults who present to the emergency department (ED) often live with several chronic diseases and take many medications. Medication harms are a frequent reason for ED visits, and in a national US surveillance study the rate of such visits differed markedly by age group and drug class [1]. Polypharmacy, most often defined as the regular use of five or more medications, is now common in this population [2]. Many of the drugs that older patients take every day, including diuretics, antidepressants, antiepileptic drugs, renin–angiotensin–aldosterone system (RAAS) inhibitors and proton pump inhibitors (PPIs), interfere directly with the handling of water and electrolytes by the kidney. Because ageing reduces the capacity of the kidney to excrete water and potassium and to conserve sodium, and because these drugs are frequently combined, older adults have a narrow margin before a drug effect becomes a clinically relevant electrolyte disturbance (Section 3). This vulnerability is reflected in the frequency of electrolyte disorders among older patients attending the ED.

Electrolyte disturbances are among the most frequent laboratory abnormalities in unselected ED patients, with hyponatremia being the most common [3]. Medication is an important contributor. In a single-center cross-sectional analysis of 22,239 ED patients (mean age 52 years), the 2514 patients taking diuretics more often had hyponatremia (20% vs. 7.7% of non-users), hypokalemia (17% vs. 11%) and hyperkalemia (13% vs. 4%) [4]. These associations differed by diuretic class: in multivariable analysis, thiazides were associated with hyponatremia (odds ratio 1.44) and hypokalemia (odds ratio 2.18) but with a lower risk of hyperkalemia (odds ratio 0.65), whereas aldosterone antagonists were associated with hyponatremia (odds ratio 2.4) and hyperkalemia (odds ratio 1.76), and amiloride with hyperkalemia (odds ratio 3.3) [4]. Because of the cross-sectional design and the incomplete recording of concomitant medications, these figures reflect an association rather than an effect of diuretics alone [4]. In a recent Spanish study of patients attending the ED with drug-induced hyponatremia, the mean age was 80.5 years and 78% were women [5]. These figures suggest that the typical patient with a drug-associated electrolyte disturbance in the ED is an older adult, frequently a woman, taking several medications.

Despite this, the role of drugs is easily overlooked in the ED. Symptoms are non-specific, medication lists are often incomplete, and the link between a recently started drug and the presenting complaint may not be obvious [6]. Most reviews of drug-induced electrolyte disorders focus on a single electrolyte or a single drug class. In older ED patients, however, polypharmacy, prescribing cascades and overlapping drug effects often produce more than one disturbance at the same time, for example hyponatremia with hypokalemia during thiazide treatment, or hypomagnesemia with secondary hypokalemia and hypocalcemia when a PPI is combined with a diuretic (Section 4, Section 5 and Section 6). Sodium, potassium, magnesium and calcium therefore need to be assessed together. This narrative review takes the perspective of the emergency physician caring for an older patient and proposes such an integrated approach. We summarize why older adults are vulnerable, which drugs are most often implicated in disorders of sodium, potassium, magnesium and calcium, how these disorders present, and how the ED can contribute to their recognition and prevention.

## 2. Materials and Methods

### 2.1. Search Strategy

We conducted a narrative review of the literature on drug-associated electrolyte disturbances in older adults, with emphasis on emergency and acute hospital settings. An initial literature search was performed on 3 October 2026. The search was subsequently re-run on 27 October 2026 in PubMed/MEDLINE with the final, fully documented search string, for English-language articles published between 1 January 2016 and 30 September 2026. The publication period was closed at 30 September 2026, the last complete month before the initial search, and the search was not updated further. Search terms combined three concepts: (i) electrolyte disorders (hyponatremia, hypernatremia, hyperkalemia, hypokalemia, hypomagnesemia, hypocalcemia and hypercalcemia); (ii) drug exposure (adverse drug event, drug-induced, drug-associated, diuretics, thiazides, antidepressants, antiepileptic drugs, antipsychotics, proton pump inhibitors, angiotensin-converting enzyme inhibitors, angiotensin receptor blockers, mineralocorticoid receptor antagonists, non-steroidal anti-inflammatory drugs, trimethoprim, desmopressin, lithium and denosumab); and (iii) population or setting (older adults, elderly, emergency department and hospitalization). Medical Subject Headings (MeSH) were combined with free-text terms in titles and abstracts; the complete search string is provided in Supplementary Table S1. Because the review focused on clinical evidence, PubMed/MEDLINE was used for the primary search, and coverage was extended by manual screening of the reference lists of included articles and recent reviews.

### 2.2. Study Selection

The search identified 1742 records. Titles and abstracts were screened independently by two authors (A.C.B. and C.S.D.), and potentially relevant articles were assessed in full text by the same two authors. Disagreements at either stage were resolved by discussion and, when consensus was not reached, by a third author (M.L.M.). Articles were eligible if they reported the frequency, risk, mechanism, clinical presentation, management or prevention of a drug-associated electrolyte disturbance in adults aged 65 years or older, or in emergency or hospital populations in which older adults represented the majority. In line with the narrative design, articles were selected for their relevance and methodological quality rather than through a formal systematic review process, and a total of 63 references were cited.

The following hierarchy was applied: (1) clinical practice guidelines, consensus statements and explicit prescribing criteria; (2) randomized controlled trials and their meta-analyses; (3) large population-based and pharmaco-epidemiological studies (cohort and case–control designs); (4) ED and hospital cohorts; and (5) narrative and systematic reviews, used mainly for mechanistic background. Case reports and small case series were excluded, as were studies limited to pediatric populations, conference abstracts and articles without full text. Guidelines and prescribing criteria were identified through the database search and the screening of reference lists; when a guideline had been updated, only the most recent version was cited.

Studies published before 2016 (“landmark studies”) were included only when they were the primary source of a quantitative estimate used in this review and no more recent study provided equivalent data in a comparable population. This applied to four sources: a cross-sectional analysis of diuretic-associated electrolyte disorders in the ED, a population-based case–control study of PPIs and hypomagnesemia, a Cochrane diagnostic review of dehydration in older people and a mechanistic review of drug-induced hypernatremia.

### 2.3. Synthesis

Because this is a narrative review, no formal risk-of-bias assessment or quantitative synthesis was performed. Throughout the text, the design of each key study is stated, and observational associations are distinguished from causal effects demonstrated in randomized trials and from recommendations in clinical guidelines. The term “drug-associated” is used for associations derived from observational data, whereas “drug-induced” is reserved for disturbances supported by an established pharmacological mechanism and a consistent temporal relationship with drug initiation, or demonstrated in randomized trials; “drug-induced” is retained in the title and section headings in its conventional pharmacovigilance sense. The strength of the evidence, the main sources of bias and the contribution of comorbidities, acute illness and concomitant medications are discussed in Section 10.

### 2.4. Use of Generative Artificial Intelligence

Generative artificial intelligence (Claude Opus 5.5, Anthropic PBC, San Francisco, CA, USA) was used only as an auxiliary tool, to assist with the formulation of search terms, the organization of the retrieved literature, and the drafting and language editing of the text. It was not used to select the final studies or to formulate clinical recommendations. All cited references were retrieved and read in full text by the authors, and all extracted data, including every numerical value reported in the manuscript, were independently verified by the authors against the original sources. Study selection and clinical interpretation were performed by the authors, who take full responsibility for the content of the manuscript.

## 3. Why Older Adults Are Vulnerable

### 3.1. Age-Related Changes in Water and Electrolyte Handling

Kidney function declines with age even in healthy individuals. In carefully screened kidney donors, the glomerular filtration rate (GFR) falls by about 6.3 mL/min/1.73 m^2^ per decade, and older adults have less renal reserve when an additional insult occurs [7]. A lower GFR limits the excretion of free water and of potassium, and leaves less room before a drug that acts on the nephron produces a clinically relevant disturbance.

The tubule also changes. Basal renin activity and aldosterone levels decrease with age. Together with interstitial fibrosis and a blunted response to natriuretic peptides, this impairs the ability of the kidney to match urinary sodium loss to intake [8]. Older adults therefore become volume depleted more easily during intercurrent illness. Na^+^/K^+^-ATPase activity also declines, which reduces the kaliuretic response. The risk of hyperkalemia is therefore higher with potassium-sparing diuretics, β-blockers and ACE inhibitors, while diuretic-induced hypokalemia is also more common in older patients [8]. The capacity to concentrate and dilute the urine falls. In one comparison, the mean maximum urine osmolality was 1109 mOsm/kg in people younger than 40 years and 882 mOsm/kg in those older than 60 years, while the minimum urine osmolality rose from 52 to 92 mOsm/kg [8]. At the same time, the thirst response weakens, which increases the risk of hypernatremia [8]. In practical terms, the older kidney tolerates neither a water load nor a water deficit well, and it handles a potassium load poorly. Each of these limits is the target of a commonly prescribed drug class.

Several other changes favor hyponatremia in particular. Aging is associated with altered regulation of vasopressin rather than a simple, uniform increase in its secretion: higher plasma vasopressin concentrations and an increased sensitivity to osmotic stimuli have been reported in older adults, and a reduced intrarenal generation of prostaglandins further limits free-water excretion [9]. The syndrome of inappropriate antidiuresis (SIAD), including its idiopathic form, is more common. And because total body water makes up a smaller proportion of body weight, a given water gain produces a larger fall in serum sodium [9]. Low solute intake, as in the “tea and toast” diet, further limits water excretion [9]. As a result, hyponatremia is found in about 11.6% of community-dwelling people aged 75 years or older, in roughly 18% of nursing home residents and in about one-third of hospitalized patients older than 65 years [9].

The same pattern is visible in the ED. The EDEN-16 study included 13,368 patients aged 65 years or older attending 52 Spanish EDs during one week. Among those with a sodium measurement, hyponatremia was present in 13.5% and hypernatremia in 2.9% [10]. Both disorders were associated with hospitalization, prolonged ED stay and in-hospital death, and the risk rose steeply with severity. Compared with normonatremic patients, the odds ratio for death was 3.07 at a sodium of 130 mmol/L and 6.34 at 120 mmol/L. For hypernatremia, it was 2.77 at 150 mmol/L and 6.33 at 160 mmol/L [10].

### 3.2. Pharmacokinetic Changes and Polypharmacy

Aging changes how drugs are absorbed, distributed and eliminated, and how target organs respond to them [11]. Drugs and active metabolites cleared by the kidney accumulate as GFR declines. A dose that was appropriate at initiation may therefore become excessive after an episode of dehydration or acute kidney injury. Aging also affects vision, swallowing, and motor and cognitive function, which makes errors in drug intake more likely [11].

Most older ED patients have several chronic conditions, each treated according to its own guideline. Polypharmacy, as defined in Section 1, is the rule rather than the exception. The most recent wave of the Survey of Health, Ageing and Retirement in Europe included 40,795 adults aged 65 years or older. The prevalence of polypharmacy was 36.2%, ranging from 25.0% to 51.8% between countries [12]. It increased with age, from 30.0% at 65–74 years to 41.1% at 75–84 years and 49.4% at 85 years or older [12]. The definition used in that survey included over-the-counter drugs and dietary supplements, which are often missing from the medication lists available in the ED.

With polypharmacy, drugs with individually small effects on electrolyte balance are combined, and the combination matters more than any single agent. In a drug surveillance program of psychiatric inpatients, the risk of hyponatremia was 16 to 42 times higher when psychotropic drugs were combined with other hyponatremia-inducing agents. These included diuretics, angiotensin-converting enzyme (ACE) inhibitors and angiotensin II receptor blockers [13]. Women aged 65 years or older taking a serotonin–norepinephrine reuptake inhibitor (SNRI) together with other such drugs formed the highest-risk subgroup [13]. Similarly, in older adults treated for urinary tract infection, the excess risk of hyperkalemia associated with trimethoprim appeared to be more pronounced among patients already taking RAAS inhibitors or spironolactone, suggesting a contribution from drug interactions [14]. Polypharmacy is also a marker of poor outcome after the ED visit. In patients attending the ED with drug-induced hyponatremia, taking more than ten medications was the only factor independently associated with a return visit within 30 days [5].

Polypharmacy also generates prescribing cascades, defined as the treatment of an adverse drug reaction with another drug [15]. Electrolyte disturbances are often part of them. A classic example starts with a dihydropyridine calcium channel blocker that causes ankle edema. The edema is treated with a diuretic, the diuretic causes hypokalemia, and the hypokalemia is in turn treated with a potassium-sparing diuretic [15]. In a US study, about 1 in 22 patients taking such calcium channel blockers also received a diuretic for edema [15]. In the same way, fatigue, confusion or unsteadiness caused by drug-induced hyponatremia may be attributed to aging, depression or dementia, and treated with drugs that lower sodium further. The ED is often the first place where the full sequence of prescriptions can be reconstructed.

Explicit criteria for potentially inappropriate prescribing in older adults address these risks directly. The 2023 American Geriatrics Society Beers Criteria recommend caution with drugs that may cause or exacerbate SIAD or hyponatremia. These include selected antidepressants (selective serotonin reuptake inhibitors [SSRIs], SNRIs, tricyclic antidepressants and mirtazapine), carbamazepine and oxcarbazepine, antipsychotics, diuretics and tramadol. The criteria advise monitoring sodium closely when these drugs are started or their dose is changed [16]. Because of the risk of hyperkalemia, the same criteria advise against routinely combining two or more RAAS inhibitors, or a RAAS inhibitor with a potassium-sparing diuretic. They also recommend caution with trimethoprim–sulfamethoxazole in patients taking ACE inhibitors, angiotensin receptor blockers or angiotensin receptor–neprilysin inhibitors when creatinine clearance is reduced [16]. The third version of the STOPP/START criteria contains 190 criteria, 133 of which describe potentially inappropriate prescriptions [17]. Although these tools were developed mainly for primary care and hospital wards, they can be used in the ED as rapid screening aids during the initial medication reconciliation, to identify potentially inappropriate medications and drug combinations that may have contributed to an acute electrolyte disturbance.

### 3.3. Frailty and the Limits of Clinical Assessment

In older adults, the clinical assessment of hydration and volume status is particularly unreliable. A Cochrane review assessed 67 clinical signs, symptoms and tests in people aged 65 years or older and found that none was consistently useful for detecting current water-loss dehydration. Commonly used measures such as dry mouth, thirst, heart rate, urine color and urine specific gravity were not useful on their own [18]. This has direct consequences for drug-induced electrolyte disturbances. Whether the patient needs isotonic saline or fluid restriction depends on distinguishing diuretic-induced hypovolemic hyponatremia from drug-induced SIAD, and that distinction rests largely on the assessment of extracellular volume [19,20]. Bedside ultrasound may provide a more objective complement to clinical examination, although the supporting evidence is still limited (Section 8.3). Frailty adds a further layer of vulnerability. Frail older adults have less physiological reserve and more often present atypically, so a drug-induced electrolyte disturbance may show up only as a decline in mobility or a fall. Chronic hyponatremia contributes to falls through gait instability and neurocognitive deficits. Even mild hyponatremia has been independently associated with a two- to three-fold higher occurrence of fractures [21]. Because the ED is often the first point of contact after such an event, it is also an opportunity to recognize the role of medication before the next prescription is added.

## 4. Drug-Induced Hyponatremia

**Figure 1:**
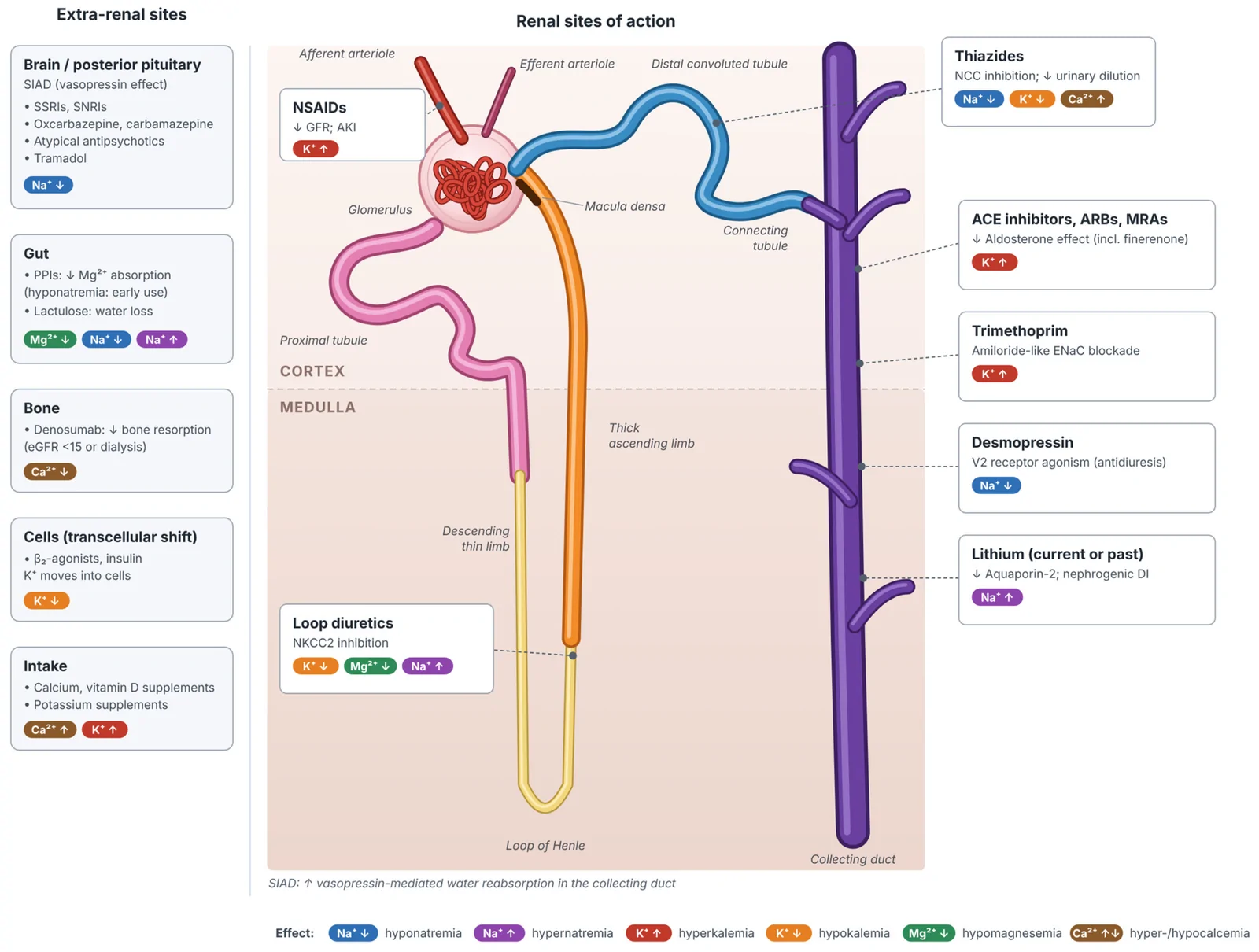
Main drug classes associated with electrolyte disturbances in older adults and their principal renal and extra-renal sites of action. Colored labels indicate the resulting disturbance. ACE, angiotensin-converting enzyme; AKI, acute kidney injury; ARBs, angiotensin II receptor blockers; DI, diabetes insipidus; eGFR, estimated glomerular filtration rate; ENaC, epithelial sodium channel; GFR, glomerular filtration rate; MRAs, mineralocorticoid receptor antagonists; NCC, sodium–chloride cotransporter; NKCC2, sodium–potassium–2-chloride cotransporter; NSAIDs, non-steroidal anti-inflammatory drugs; PPIs, proton pump inhibitors; SIAD, syndrome of inappropriate antidiuresis; SNRIs, serotonin–norepinephrine reuptake inhibitors; SSRIs, selective serotonin reuptake inhibitors; V2, vasopressin type 2 receptor.

Drugs cause hyponatremia through two main mechanisms: loss of sodium, typically with diuretics, and impaired excretion of free water, most often through SIAD or an increased renal sensitivity to vasopressin [6,20]. Thiazides act in the distal convoluted tubule, a key site of urinary dilution; antidepressants increase vasopressin levels or the renal response to vasopressin; antipsychotics may increase vasopressin release; and carbamazepine and oxcarbazepine enhance collecting duct water permeability through the vasopressin V2 receptor–aquaporin-2 pathway [6,20]. In a pharmacovigilance program cited in a recent review, about 70% of severe drug-induced cases were hypovolemic and mostly related to diuretics, while about 30% had the features of SIAD [6]. The main drugs, their mechanisms and the period of highest risk are summarized in Table 1, and their principal sites of action are shown in Figure 1.

### 4.1. Thiazide Diuretics

Thiazides remain the single most important cause of drug-induced hyponatremia. They impair urinary dilution in the distal convoluted tubule, so that even a modest water intake can lower serum sodium. In a Swedish population-based study, thiazides accounted for about 27% of hospitalizations due to hyponatremia [22]. The risk appeared highest at the start of treatment: the adjusted odds ratio for hospitalization was 48 in the first week after initiation and fell to 2.9 in long-term users [22]. The absolute risk, however, was low, at about 0.12% of newly treated patients [22].

A 2026 cohort study clarified who carries this risk. Among 159,080 adults starting either a thiazide or a calcium channel blocker, the two-year incidence of profound hyponatremia (sodium below 125 mmol/L) was 0.80% with thiazides and 0.46% with calcium channel blockers [23]. In thiazide users, the incidence rose from 0.40% under the age of 65 years to 1.05% at 65–79 years and 2.40% at 80 years or older, and it was consistently higher in women. In women aged 80 years or older it reached 3.06%, corresponding to one case of profound hyponatremia for every 53 women treated [23]. The relative risk was highest in the first 14 days.

The excess risk in women is not limited to thiazides. In a Dutch nested case–control study of 2506 diuretic users admitted to hospital, in which the mean age of patients with hyponatremia was 77.9 years, women had higher odds of hyponatremia on admission than men with thiazides (adjusted odds ratio 1.35) and with loop diuretics (adjusted odds ratio 1.62), and almost twice the odds of severe hyponatremia (adjusted odds ratio 1.96) [35].

Other antihypertensive drugs carry a much smaller risk. In a Swedish case–control study of patients hospitalized with severe hyponatremia, ongoing treatment with ACE inhibitors, angiotensin II receptor blockers, beta-blockers or calcium channel blockers was not associated with an increased risk, whereas treatment started within the previous 90 days was associated with a modest increase, with adjusted odds ratios up to 2.24 for ACE inhibitors [36]. In the ED, this means that a new antihypertensive drug deserves attention, but a thiazide, including one hidden in a fixed-dose combination, is a considerably more likely contributor. In the Spanish ED cohort, hydrochlorothiazide was involved in half of all drug exposures implicated in hyponatremia [5]. In an older woman who presents with confusion, a fall or vomiting a few weeks after starting a thiazide, thiazide-associated hyponatremia should be considered a leading possibility, while other causes of the presentation are evaluated in parallel.

### 4.2. Antidepressants and Antiepileptic Drugs

SSRIs and SNRIs cause hyponatremia mainly through SIAD [6,20]. They should not be considered separately from thiazides: the two classes act through complementary mechanisms, thiazides by impairing urinary dilution and causing sodium loss and antidepressants by promoting water retention, and they are frequently co-prescribed in older adults. Their combination therefore represents a compounding risk factor; in a large drug surveillance program, the risk of hyponatremia was 16 to 42 times higher when psychotropic drugs were combined with other hyponatremia-inducing agents, including diuretics [13]. The risk with SSRIs also follows the same time course as that of thiazides. In a population-based study, the adjusted odds ratio for hospitalization due to hyponatremia was 29 in the first week after starting an SSRI and declined to 2.1 by week 13, whereas patients on long-term treatment were not at increased risk [25]. In a large drug surveillance program, hyponatremia typically appeared a median of 7 days after the first dose or a dose increase [13]. Among antidepressants, mirtazapine appears to carry a lower risk [6].

Among antiepileptic drugs, oxcarbazepine carries the highest risk, followed by carbamazepine. In the same surveillance program, hyponatremia occurred in 1.66% of patients treated with oxcarbazepine and 0.17% of those treated with carbamazepine [13]. Unlike the antidepressants, the risk with these drugs does not disappear with time, and it increases with treatment duration [6]. Combinations matter: in the same program, the risk was considerably higher when several psychotropic drugs that can cause hyponatremia were taken together [13]. In the Spanish ED cohort, antidepressants and antiepileptic drugs were the most frequently implicated groups after hydrochlorothiazide, accounting for 15.2% and 7.6% of drug exposures, respectively [5].

Antipsychotics are widely used in older adults for delirium, behavioral symptoms of dementia and psychosis. In a population-based cohort of 58,008 matched pairs of adults aged 65 years or older (mean age 81 years) newly treated with risperidone, olanzapine or quetiapine, hospitalization with hyponatremia within 30 days occurred in 0.15% of users and 0.09% of non-users (relative risk 1.62) [26]. The absolute increase was small (0.06%) and lower than that reported for other psychotropic drugs [26]. The 2023 AGS Beers Criteria nevertheless include antipsychotics among the drugs that may cause or worsen SIAD or hyponatremia in older adults and recommend close monitoring of sodium when they are started or their dose is changed [16].

### 4.3. Proton Pump Inhibitors and Desmopressin

PPIs are among the most widely prescribed drugs in older adults. Beyond their well-known effect on magnesium (Section 6.1), they have been associated with hospitalization due to hyponatremia. In a Swedish case–control study of 11,213 patients hospitalized with hyponatremia (mean age 76 years, 72% women), the adjusted odds ratio for omeprazole or esomeprazole was 6.87 in the first week after initiation, declined over the following weeks and was only 1.10 with ongoing treatment [27]. The mechanism remains incompletely understood. PPIs have been listed among drugs associated with SIAD and, less often, with interstitial nephritis and renal salt loss [6], whereas the early peak of risk may also partly reflect confounding by indication, which the authors could not exclude [27].

Desmopressin, used in older adults for nocturia, is a synthetic analogue of vasopressin that activates V2 receptors in the renal collecting duct, increasing aquaporin-2-mediated free-water reabsorption; when fluid intake is not reduced accordingly, the resulting water retention leads to dilutional hyponatremia [20]. In a cohort of 3137 adults with a mean age of 70 years who started desmopressin, the rate of hyponatremia was 146 per 1000 person-years, compared with 11 per 1000 person-years in matched patients starting oxybutynin, an adjusted hazard ratio of 13.2 [31]. Desmopressin should therefore always be specifically asked about in an older patient presenting with hyponatremia, because it may not be perceived as a ‘real’ medication by the patient or the family.

Tramadol is commonly used for pain in older adults. The 2023 AGS Beers Criteria advise caution with tramadol because it may cause or worsen SIAD or hyponatremia, and recommend close monitoring of sodium when it is started or its dose is changed [16]. When tramadol is taken together with an antidepressant or a diuretic, it should be considered a possible contributor even if another drug seems to be the obvious cause.

### 4.4. Implications for Treatment in the Emergency Department

Drug-induced hyponatremia has a specific implication for treatment. Once the offending drug is stopped, the stimulus for water retention may disappear quickly, and a brisk water diuresis can raise serum sodium faster than intended [19,20]. This is particularly relevant after a thiazide is withdrawn or volume is restored in a patient with diuretic-induced hypovolemia. Older patients with chronic hyponatremia, low solute intake or hypokalemia are those in whom overly rapid correction is most likely and most harmful, so sodium should be monitored closely after the drug is stopped [19]. Distinguishing diuretic-induced hypovolemia from SIAD at the bedside is often difficult in older patients; the contribution of point-of-care ultrasound (POCUS) to this assessment is discussed in Section 8.3.

### 4.5. Drug-Associated Hypernatremia

Hypernatremia is less common than hyponatremia in older ED patients, but its prognosis is similarly poor. In the EDEN-16 study it was present in 2.9% of patients aged 65 years or older with a sodium measurement, and the odds ratio for death was 2.77 at a sodium of 150 mmol/L and 6.33 at 160 mmol/L [10]. In a Swedish cohort, infections caused 51% of hypernatremia episodes in patients aged 65 years or older, and 25% of all episodes were fatal [37]. In older adults, drugs usually act as contributors to hypernatremia rather than as its sole cause, and their effect is most dangerous when thirst is impaired or access to water is limited [38].

Several mechanisms are involved [38]. Lithium is the drug most frequently reported to cause hypernatremia; it reduces the expression of aquaporin-2 water channels in the collecting duct and causes nephrogenic diabetes insipidus. Loop diuretics impair urinary concentration, osmotic agents such as lactulose and mannitol cause hypotonic water loss through the gut or the kidney, and drugs that cause hypokalemia or hypercalcemia can produce an acquired form of nephrogenic diabetes insipidus. Less often, hypernatremia results from a sodium load, for example with sodium bicarbonate or hypertonic saline [38].

The effect of lithium can outlast treatment. In the Swedish cohort, nephrogenic diabetes insipidus or marked polyuria was present in 16% of hypernatremia episodes, while lithium intoxication accounted for only about 1% [37]. The odds of an episode associated with nephrogenic diabetes insipidus were increased with current lithium use (odds ratio 10.3), with past lithium use (odds ratio 6.7) and with treatment lasting five years or longer (odds ratio 9.4) [37]. Lithium-induced nephrogenic diabetes insipidus is usually reversible after discontinuation, but in some patients the urinary concentrating defect persists long after lithium has been stopped, probably reflecting sustained impairment of collecting duct function and aquaporin-2 regulation [38]. The degree of reversibility varies between patients, which explains why past as well as current lithium exposure was associated with nephrogenic diabetes insipidus in the Swedish cohort [37]. Hypernatremia itself develops only when these water losses are not matched by intake, that is, when thirst is impaired, access to water is limited or an intercurrent illness reduces intake; in older adults, the diminished osmotic stimulation of thirst further increases this risk [38]. In the ED, an older patient with hypernatremia should therefore be asked about current and previous lithium treatment, as well as laxatives and diuretics, particularly when an infection has reduced fluid intake.

## 5. Drug-Induced Disorders of Potassium

Potassium disturbances are among the most frequent laboratory abnormalities in the ED. In a cohort of 12,799 emergency patients, hypokalemia was found in 20.1% and hyperkalemia in 2.98% [39]. In a large hospital cohort from the United Kingdom, the prevalence of hyperkalemia was 8.57% in the ED and 16.79% among hospitalized patients, and higher potassium concentrations were associated with chronic kidney disease and heart failure [40]. Older adults are exposed to both ends of this spectrum, because drugs that raise potassium, such as RAAS inhibitors and mineralocorticoid receptor antagonists (MRAs), are often prescribed together with drugs that lower it, such as loop and thiazide diuretics.

The mechanisms are well defined. Potassium excretion depends on aldosterone-stimulated secretion in the distal nephron and on adequate distal sodium delivery and urine flow. Drug-associated hyperkalemia may therefore result from drugs that reduce aldosterone synthesis or action (ACE inhibitors, angiotensin II receptor blockers and MRAs), block the epithelial sodium channel (trimethoprim and potassium-sparing diuretics), or impair renin release (non-steroidal anti-inflammatory drugs [NSAIDs]) [14,32,33]. Drug-associated hypokalemia may result from increased distal sodium delivery combined with secondary hyperaldosteronism (loop and thiazide diuretics), from a shift of potassium into cells (β_2_-agonists and insulin), or from gastrointestinal losses (laxatives) [34]. Reduced GFR, heart failure and diabetes limit potassium excretion and are the main patient-level determinants of hyperkalemia, whereas vomiting, diarrhea, reduced intake and hypomagnesemia favor hypokalemia [32,33,34].

**Figure 2:**
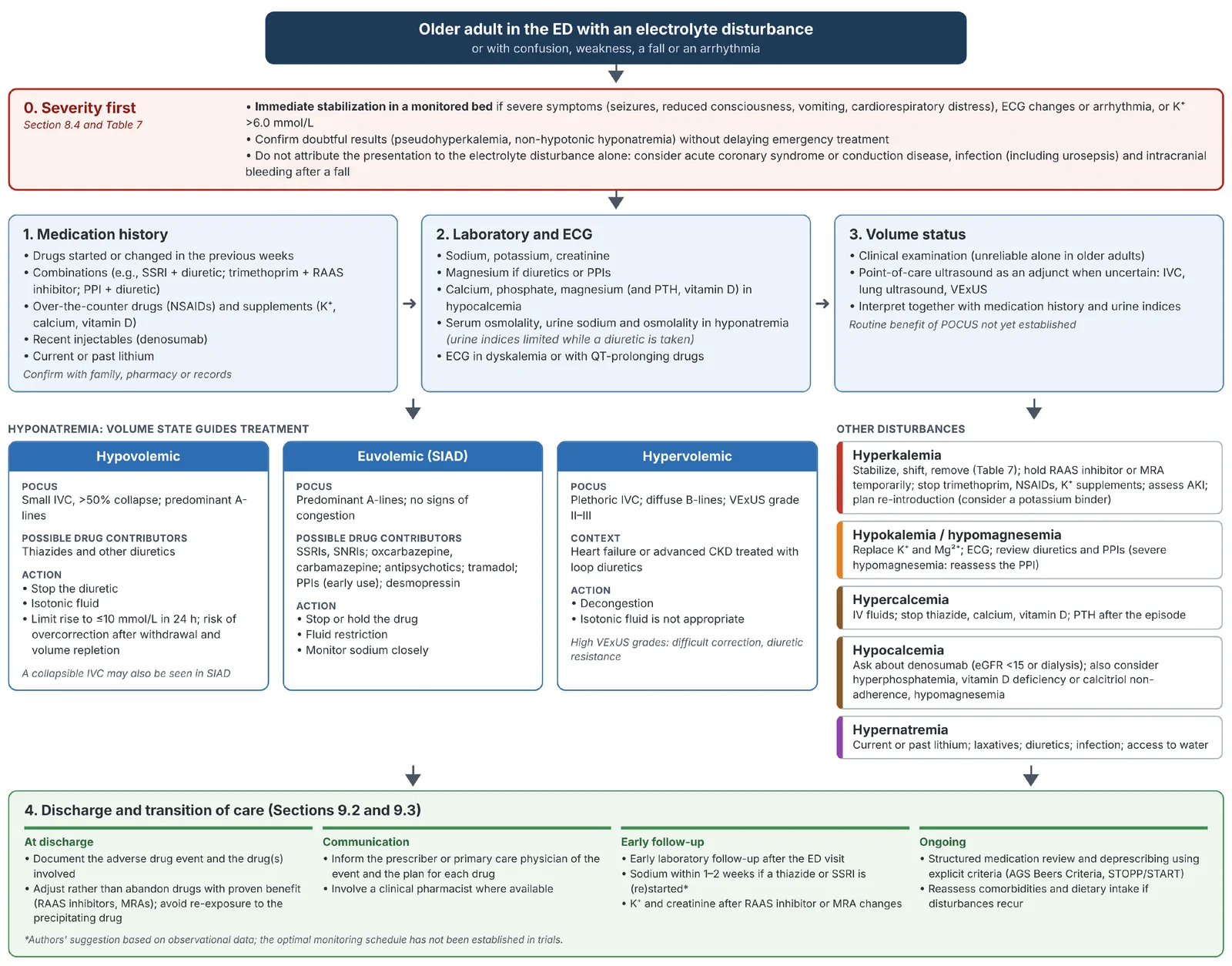
Practical, severity-based and medication-focused approach to older adults presenting to the emergency department with a suspected drug-associated electrolyte disturbance from initial assessment to transition of care. Step 0 and the management of individual disturbances are detailed in Section 8.4; step 4 is detailed in Section 9.2 and Section 9.3. AGS, American Geriatrics Society; AKI, acute kidney injury; CKD, chronic kidney disease; ECG, electrocardiogram; ED, emergency department; eGFR, estimated glomerular filtration rate; IV, intravenous; IVC, inferior vena cava; MRA, mineralocorticoid receptor antagonist; NSAIDs, non-steroidal anti-inflammatory drugs; POCUS, point-of-care ultrasound; PPI(s), proton pump inhibitor(s); PTH, parathyroid hormone; RAAS, renin–angiotensin–aldosterone system; SIAD, syndrome of inappropriate antidiuresis; SNRIs, serotonin–norepinephrine reuptake inhibitors; SSRI(s), selective serotonin reuptake inhibitor(s); STOPP/START, Screening Tool of Older Persons’ Prescriptions/Screening Tool to Alert to Right Treatment; VExUS, venous excess ultrasound.

These sites of action are illustrated in Figure 1: NSAIDs act at the level of the glomerulus and afferent arteriole, where they reduce GFR and may precipitate acute kidney injury; loop diuretics act in the thick ascending limb and thiazides in the distal convoluted tubule; ACE inhibitors, angiotensin II receptor blockers and MRAs reduce the aldosterone effect in the collecting duct, where trimethoprim blocks the epithelial sodium channel; and β_2_-agonists and insulin shift potassium into cells at extra-renal sites. The corresponding ED actions for hyperkalemia and hypokalemia are summarized in Figure 2 and Section 8.4.

### 5.1. Hyperkalemia

RAAS inhibitors and MRAs are the drugs most often associated with hyperkalemia. In patients treated with these agents, hyperkalemia is almost twice as common as in untreated patients (risk ratio 1.89) [32]. Older adults combine the main risk factors: reduced GFR, heart failure and diabetes, conditions in which these drugs are most strongly indicated [32,33]. The way hyperkalemia is handled in hospital has consequences beyond the acute episode. In the UK cohort, RAAS inhibitors were discontinued during admission in 36% of patients with potassium of 5.0–5.5 mmol/L and in 61% of those with potassium above 6.5 mmol/L; at discharge, 32% had their RAAS inhibitor stopped and only 2% had the dose reduced, and discontinuation was associated with worse survival [40]. For this reason, current recommendations favor maintaining RAAS inhibitors at evidence-based doses, with review of other contributing drugs and diet and, when needed, potassium binders, and reserve dose reduction for situations in which these measures fail [32].

Newer drugs acting on the mineralocorticoid receptor carry the same risk. In the FIDELITY pooled analysis of 13,026 patients with type 2 diabetes and chronic kidney disease, hyperkalemia occurred in 14.0% of patients treated with finerenone and 6.9% of those receiving placebo, and led to permanent discontinuation in 1.7% and 0.6%, respectively; no hyperkalemia event was fatal [41]. These rates were observed under protocol-driven potassium monitoring, which is not always achieved in routine care of older patients.

Short courses of other drugs can tip the balance. Trimethoprim has a potassium-sparing, amiloride-like effect on the distal nephron [14]. In a cohort of more than one million adults aged 65 years or older, trimethoprim prescribed for urinary tract infection was associated with a higher risk of hyperkalemia (odds ratio 2.27) and of acute kidney injury (odds ratio 1.72) compared with amoxicillin [14]. The excess risk was small in the general population but reached about 18 additional cases of hyperkalemia per 1000 treated infections in patients taking RAAS inhibitors or spironolactone [14]. An older patient on an ACE inhibitor or an MRA who presents with hyperkalemia a few days after an antibiotic prescription should therefore be asked specifically about trimethoprim or co-trimoxazole. Hyperkalemia in this setting is, however, usually multifactorial, and trimethoprim should not be assumed to be the only cause: acute kidney injury due to volume depletion during the infection, which was itself more frequent with trimethoprim [14], unreported over-the-counter NSAID use [42] and the intake of potassium supplements should be assessed at the same time.

NSAIDs are another common trigger. In a population-based study of 46,107 adults aged 66 years or older who started an NSAID and were matched to non-users, hyperkalemia within 30 days occurred in 0.40% of users compared with 0.27% of non-users (odds ratio 1.50), and acute kidney injury in 0.82% compared with 0.59% (odds ratio 1.41) [42]. The absolute risks were low, but the two outcomes are linked, because acute kidney injury itself reduces potassium excretion.

When hyperkalemia in patients taking potassium-raising drugs is severe, the outcome is often poor, although this partly reflects the underlying illness. In an ED series of 90 adults with severe hyperkalemia and a mean age of 71.1 years, in-hospital mortality was 28.9%, and spironolactone, potassium chloride and ACE inhibitors or angiotensin II receptor blockers were among the drugs recorded before presentation [43]. In the larger ED cohort, potassium above 7 mmol/L was associated with ICU admission (odds ratio 7.57), and potassium above 6.5 mmol/L with in-hospital death (odds ratio 6.25 for 6.5–7 mmol/L and 5.87 above 7 mmol/L) [39].

Not all drug effects on potassium are harmful, and some help to keep RAAS inhibitors in use. In a meta-analysis of individual participant data from six randomized trials including 49,875 people with type 2 diabetes, sodium–glucose cotransporter 2 (SGLT2) inhibitors reduced the risk of serious hyperkalemia (hazard ratio 0.84) without increasing the risk of hypokalemia [44]. In the DIAMOND trial, which included patients with heart failure with reduced ejection fraction and prior or current hyperkalemia related to RAAS inhibitors, patiromer reduced the risk of hyperkalemia above 5.5 mmol/L (hazard ratio 0.63) and of MRA dose reduction (hazard ratio 0.62) compared with placebo [45]. In the ED, the acute treatment of drug-induced hyperkalemia follows the same principles as hyperkalemia of other causes and combines three modalities: stabilization of the cardiac membrane with intravenous calcium when ECG changes are present; shifting of potassium into cells with intravenous insulin and glucose or nebulized β_2_-agonists; and removal of potassium from the body with loop diuretics, potassium binders or, when potassium remains elevated or ECG changes do not respond to medical treatment, hemodialysis [32,33] (Section 8.4); the specific contribution of the ED is to identify the drugs involved and to make sure that a temporary interruption is not converted into a permanent one without a clear decision.

### 5.2. Hypokalemia

Thiazide and loop diuretics are the main causes of drug-induced hypokalemia, through increased distal sodium delivery and aldosterone-mediated potassium secretion [34]. Among ED patients, hypokalemia was present in 17% of diuretic users compared with 11% of non-users [4]. In the community, hypokalemia is much less common. In the Polish PolSenior survey of 4654 older adults (mean age 76.5 years), hypokalemia was found in only 0.84% of participants, more often in women than in men (1.23% vs. 0.46%) [46]. Among participants with hypertension, the prevalence was 1.96% in those taking potassium-losing diuretics and 0.46% in untreated participants, potassium supplementation did not lower this prevalence, and no hypokalemia was found in participants taking spironolactone [46]. The contrast with ED data suggests that, in older patients, hypokalemia in chronic diuretic users often becomes manifest when an acute illness, such as vomiting, diarrhea or reduced food intake, is added.

Other drugs shift potassium into cells, including inhaled or intravenous β_2_-agonists and insulin, while laxative use contributes to gastrointestinal losses [34]. Hypomagnesemia, which is itself often drug-induced (Section 6.1), makes hypokalemia difficult to correct, so magnesium should be measured in every older patient with drug-induced hypokalemia [30,34].

In older patients, hypokalemia is clinically important mainly because it increases the risk of ventricular arrhythmias, particularly when QT-prolonging drugs are taken at the same time. Electrolyte disturbances are included as risk factors in the tools developed to assess the risk of QT-prolonging pharmacotherapy in older adults [47]. The benefit of treating mild hypokalemia in the ED is less clear. In a propensity-matched ED study of patients with potassium of 3.0–3.4 mmol/L, potassium replacement was not associated with lower hospital mortality (3.3% vs. 3.9%; adjusted odds ratio 0.81) [48]. The study population was relatively young, with a mean age of 53.9 years [48], and its results should not be extended to older patients taking QT-prolonging drugs, in whom correction remains reasonable. When treatment is needed, three components should be considered: oral potassium replacement, which is preferred in non-critical situations; intravenous potassium chloride with continuous ECG monitoring when potassium is below 2.5 mmol/L or cardiac complications are present, usually at 10 mmol/h and not exceeding 20 mmol/h; and correction of concomitant hypomagnesemia, together with review of the causative diuretic [33,34] (Section 8.4). The main drugs involved in potassium disturbances and the practical steps in the ED are summarized in Table 2.

## 6. Drug-Induced Disorders of Magnesium and Calcium

### 6.1. Hypomagnesemia

Long-term PPI therapy is the best-documented drug-related contributor to hypomagnesemia [29,30], and diuretics are the other main group involved [30]. The two act at different sites, which are shown in Figure 1: PPIs reduce the active intestinal absorption of magnesium (extra-renal sites, gut), whereas loop diuretics inhibit the sodium–potassium–2-chloride cotransporter (NKCC2) and reduce magnesium reabsorption in the thick ascending limb, and thiazides inhibit the sodium–chloride cotransporter (NCC) and increase urinary magnesium loss in the distal convoluted tubule [30]. The combination of a PPI with a diuretic therefore affects both intestinal absorption and renal reabsorption of magnesium, which may explain why concomitant diuretic use is the main risk factor for PPI-associated hypomagnesemia [28,30]. In a population-based case–control study of adults aged 66 years or older, current PPI use was associated with hospitalization for hypomagnesemia (adjusted odds ratio 1.43) [28]. The association was restricted to patients also taking diuretics (adjusted odds ratio 1.73), and was not significant in those who were not [28]. The absolute risk was small, but the combination of a PPI and a diuretic is very common in older adults.

Hospital data show how severe this interaction can become. Among 53,149 patients who had magnesium measured in a Singapore hospital, 360 had severe hypomagnesemia (below 0.4 mmol/L), and in 189 of them a PPI was judged to be at least a possible cause [49]. These patients had a mean age of 71.8 years. Compared with long-term PPI users without hypomagnesemia, they more often took diuretics (39.7% vs. 17.3%; adjusted odds ratio 1.68) and high-dose PPIs (adjusted odds ratio 1.96); female sex, diabetes, low body mass index and renal impairment were further risk factors [49]. The PPI was stopped in only 22.8% of cases, and hypomagnesemia recurred in 69.7% of patients who continued the PPI compared with 35.7% of those who stopped it [49]. For the ED, this means that severe hypomagnesemia in an older patient on a PPI should lead to a clear recommendation to reassess the PPI, not only to magnesium replacement.

In this review, hypomagnesemia refers to a low serum magnesium concentration (below 0.65–0.70 mmol/L), whereas magnesium depletion refers to a deficit in total body magnesium. Because magnesium is stored mainly intracellularly and exchanges slowly with the extracellular pool, depletion may be present while the serum concentration is still normal [30]. Hypomagnesemia is often missed because magnesium is not measured routinely [30]. Its main clinical relevance lies in its effects on other electrolytes and on the heart. It causes renal potassium wasting, so that hypokalemia does not respond to replacement until magnesium is corrected (Section 5.2), and it impairs parathyroid hormone (PTH) secretion and action, leading to hypocalcemia [30,34]. In an older patient with refractory hypokalemia, hypocalcemia or a prolonged QT interval, the medication list should be checked for PPIs and diuretics, and magnesium should be measured. Management combines intravenous magnesium sulfate in severe or symptomatic hypomagnesemia, oral magnesium salts in mild, asymptomatic cases, with dose reduction in chronic kidney disease, and correction of the underlying cause, including reassessment of the PPI and diuretic [30] (Section 8.4).

### 6.2. Hypercalcemia and Hypocalcemia

Severe hypercalcemia is uncommon in the ED but has a poor prognosis. In a Swiss ED cohort of 31,963 patients with calcium measured on admission, hypercalcemia (above 2.60 mmol/L) was found in 2.7%, and 161 patients, with a mean age of 69 years, had severe hypercalcemia (2.80 mmol/L or higher) [50]. Malignancy was the most common identifiable cause (40%), followed by primary hyperparathyroidism (22%); 18% of the patients were taking thiazide-like diuretics, vitamin D and calcium supplements were also frequently recorded, and in-hospital mortality was 16% [50].

By inhibiting NCC in the distal convoluted tubule, thiazides increase calcium reabsorption and reduce urinary calcium excretion (Figure 1), and can raise serum calcium on their own or in combination with calcium supplements [24,50]. In a population-based study of 221 patients with thiazide-associated hypercalcemia, the mean age was 67 years and 86.4% were women; the incidence was 12 per 100,000 over the study period, and primary hyperparathyroidism was subsequently diagnosed in 24% [24]. Thiazide-associated hypercalcemia is therefore not only a drug effect: in about one in four patients it unmasks primary hyperparathyroidism, and PTH should be measured once the acute episode has been treated. In older adults, calcium and vitamin D are often taken as over-the-counter products and may not appear on the medication list unless the patient is asked specifically. In the ED, treatment of hypercalcemia combines intravenous fluids, withdrawal of thiazides and calcium or vitamin D supplements, and, in selected patients with severe hypercalcemia, bisphosphonates, calcitonin or loop diuretics, which were used in 16%, 11% and 16% of patients, respectively, in the Swiss ED cohort [50] (Section 8.4). Symptomatic hypocalcemia requires intravenous calcium with ECG monitoring, correction of concomitant hypomagnesemia, and subsequent oral calcium and vitamin D, with serial calcium measurements until stable (Section 8.4).

Denosumab, increasingly used for osteoporosis, has been associated with severe hypocalcemia in older adults with advanced kidney disease. By inhibiting the receptor activator of nuclear factor κB ligand (RANKL), it suppresses osteoclast-mediated bone resorption and thus the release of calcium from bone [51]. In a population-based cohort of 59,151 adults older than 65 years (mean age 79.3 years) who started denosumab, mild hypocalcemia (albumin-corrected calcium below 2.00 mmol/L) within 180 days occurred in 0.6%, compared with 0.3% of those starting an oral bisphosphonate [51]. The risk depended strongly on kidney function: it was 0.36% with an estimated GFR (eGFR) of 60 mL/min/1.73 m^2^ or higher, 0.69% at 30–59, 3.5% at 15–29 and 24% in patients with an eGFR below 15 or on dialysis, in whom severe hypocalcemia (below 1.80 mmol/L) occurred in 14.9% [51]. Only about one third of denosumab users had calcium measured within 180 days [51]. Severe hypocalcemia in an older patient with advanced chronic kidney disease or on dialysis should therefore prompt a specific question about a recent denosumab injection, which is usually given in an outpatient setting and is easily omitted from the medication history. Denosumab should not, however, be assumed to be the only cause. In advanced chronic kidney disease, hyperphosphatemia, vitamin D deficiency or non-adherence to calcitriol or other active vitamin D analogues, and hypomagnesemia, which impairs PTH secretion and action (Section 6.1) [30], are common alternative or concurrent causes. Phosphate and magnesium, and when available PTH and vitamin D status, should therefore be assessed at the same time, to avoid premature diagnostic closure.

### 6.3. Patient-Level Risk Factors and Monitoring

Across drug classes, whether a drug produces a clinically relevant disturbance depends largely on patient characteristics. Older age and female sex increase the risk of thiazide- and diuretic-associated hyponatremia [23,35]; reduced GFR, heart failure and diabetes are the main determinants of hyperkalemia with RAAS inhibitors and MRAs [32,33,40]; and female sex, diabetes, low body mass index and renal impairment were associated with severe PPI-associated hypomagnesemia [49]. Advanced chronic kidney disease is the main determinant of denosumab-associated hypocalcemia [51], and impaired thirst or restricted access to water determines whether lithium-induced nephrogenic diabetes insipidus leads to hypernatremia [37,38]. Frailty, low solute intake and acute illness reduce the margin before any of these drugs produces a clinically relevant disturbance (Section 3.1 and Section 3.3) [9], and concomitant medications frequently act as the trigger (Section 3.2). Table 3 links the main drug classes with their mechanisms, patient-level risk factors, typical presentation and suggested monitoring, and indicates whether each monitoring step is recommended by current guidance or suggested on the basis of observational data.

## 7. Clinical Presentation and Consequences in the Emergency Department

### 7.1. Clinical Presentation

Drug-induced electrolyte disturbances rarely present with a specific complaint. In older adults they typically appear as confusion, weakness, nausea, gait instability or a fall, and they are often discovered on routine blood tests [6,9]. The symptoms are easily attributed to age, dementia or an intercurrent infection, which is one reason why the medication list is not always reviewed.

Falls are the most important of these presentations. In a French emergency geriatric unit, 696 patients aged 75 years or older (mean age 86.1 years) admitted for non-vital emergencies were studied; mild hyponatremia (sodium 130–135 mmol/L) was present in 15.9% overall, in 26.1% of patients admitted for a fall and in 13.2% of the others, and it remained associated with falls after adjustment (adjusted odds ratio 3.02) [52]. Hyponatremia is also associated with reduced bone mineral density and with fractures [21]. In older ED patients, a fall may therefore be associated with, or represent an early manifestation of, a drug-associated electrolyte disturbance, and sodium should be checked in every older patient presenting after a fall who takes a thiazide, an antidepressant or an antiepileptic drug.

Electrolyte disturbances or suspected drug effects should not, however, be assumed to fully explain a fall or altered mental status. In older ED patients, these presentations frequently have other or concurrent causes, including acute coronary syndrome or conduction disturbances, systemic infection, including atypical presentations of urosepsis, and acute intracranial pathology such as subdural hematoma after a fall, which should be evaluated in parallel to avoid premature diagnostic closure (Figure 2, step 0).

The other clinically important presentations are cardiac. Hypokalemia and hypomagnesemia increase the risk of ventricular arrhythmias in patients taking QT-prolonging drugs (Section 5.2 and Section 6.1), and severe hyperkalemia may present with bradycardia, conduction disturbances or cardiac arrest [33]. For this reason, an electrocardiogram (ECG) is part of the initial assessment of every older patient with a potassium or magnesium disturbance (Section 8). When a fall, weakness or confusion is judged to be at least partly related to an electrolyte disturbance, management follows the severity of the presentation (Section 8.4): immediate, controlled intravenous correction or fluid management in patients with severe symptoms or ECG changes; temporary withholding of the suspected drug, with consideration of a safer alternative; and discharge planning that includes medication review, with involvement of a clinical pharmacist where available, and structured deprescribing to reduce the risk of recurrence (Section 9).

### 7.2. Prognosis and Recurrence

The prognostic impact of these disturbances is substantial, whatever their cause. Among 57,427 ED patients, the adjusted odds ratio for in-hospital death rose from 2.39 in mild hyponatremia to 6.98 in severe hyponatremia, compared with normonatremic patients [53]. In older ED patients the gradient is similar: in the EDEN-16 study, the odds ratio for death compared with normonatremic patients was 3.07 at a sodium of 130 mmol/L and 6.34 at 120 mmol/L [10]. Among 47,089 ED patients with potassium measured, hypokalemia was present in 5.5% and hyperkalemia in 3.6%; both were associated with admission and death, and the adjusted odds ratio for death reached 5.0 when potassium exceeded 6.0 mmol/L [54]. Comparable gradients have been reported for hyperkalemia in a more recent ED cohort [39], and in-hospital mortality reached 16% among ED patients with severe hypercalcemia [50].

Drug exposure is common among the patients with the worst outcomes. In a hospital cohort of 17,693 inpatients aged 80 years or older (mean age 84.6 years), hyponatremia was present in 24.7%; among patients with hyponatremia, 59.7% were taking PPIs and 57.4% loop diuretics, and in-hospital mortality increased from 8.9% in mild to 15.6% in moderate and 20.7% in severe hyponatremia [55]. In the ED cohort of diuretic users, all forms of dysnatremia and dyskalemia were independent risk factors for in-hospital mortality [4]. Whether these associations reflect the electrolyte disturbance itself or the underlying illness and frailty cannot be determined from observational data, but they identify a group of patients who need careful assessment.

Drug-associated disturbances also tend to recur. In the Spanish ED cohort, about 11% of patients with drug-induced hyponatremia returned to the ED with hyponatremia within 30 days [5]. Treatment changes at discharge were associated with revisits in univariable analysis, which suggests that the ED visit is a missed opportunity for medication review in many patients [5]. The same applies to hypomagnesemia: severe PPI-associated hypomagnesemia recurred in 69.7% of patients who continued the PPI, compared with 35.7% of those who stopped it [49]. The key studies on drug-induced electrolyte disturbances in older adults are summarized in Table 4.

## 8. Recognition in the Emergency Department: A Medication-Focused Approach

### 8.1. Medication History

The most important step in recognizing a drug-induced electrolyte disturbance is to ask the right question about medications. Two features of the evidence reviewed above are particularly useful. First, for thiazides, SSRIs, PPIs and desmopressin, the risk observed in population-based studies was highest in the first weeks after treatment was started or the dose was increased [22,23,25,27,31]. The question ‘Has any medication been started or changed in the last two months?’ is therefore more informative than a simple list of current drugs. Second, the risk is often driven by combinations, such as an SSRI with a diuretic, trimethoprim with a RAAS inhibitor, or a PPI with a diuretic [13,14,28,49].

Obtaining an accurate medication history in the ED is difficult. In a Danish ED study of 100 patients (median age 66.5 years), 79% had at least one discrepancy between the shared electronic medication record and the drugs they actually took; the most common type was a drug that was recorded but no longer used, and drugs for acid-related disorders and agents acting on the renin–angiotensin system were among the groups most often involved [56]. Drugs that patients or families may not report spontaneously, such as desmopressin, over-the-counter PPIs or NSAIDs, laxatives and calcium or vitamin D supplements, should be asked about explicitly, and recent injectable treatments, such as denosumab, should also be considered.

Structured programs can help, although their effect on hard outcomes is uncertain. In a systematic review of 25 studies including 44,640 older ED patients, clinical pharmacist review was associated with more deprescribing of potentially inappropriate medications (odds ratio 0.68) but not with fewer hospital admissions or a shorter length of stay, while computerized clinical decision support reduced the ordering of potentially inappropriate medications (odds ratio 0.60) [57].

### 8.2. Laboratory and ECG Assessment

Laboratory and ECG assessment should be adapted to the medications identified. In a patient taking diuretics or PPIs, magnesium should be measured together with sodium and potassium [28,30]. Calcium should be measured in patients with advanced chronic kidney disease who have recently received denosumab [51]. Urine sodium and osmolality help to separate diuretic-induced sodium loss from SIAD, but their interpretation is limited while the patient is still taking a diuretic [19,20]. An ECG is indicated in patients with dyskalemia and in those taking QT-prolonging drugs; electrolyte disturbances are among the risk factors included in the tools used to assess the risk of QT-prolonging pharmacotherapy in older adults [47]. A practical sequence for the ED is proposed in Table 5.

### 8.3. Point-of-Care Ultrasound in the Assessment of Volume Status

The distinction between hypovolemic, euvolemic and hypervolemic hyponatremia determines treatment, but it is difficult at the bedside, particularly in older adults. A Cochrane review found that no single symptom, sign or test, and no combination of them, was clearly useful for identifying water-loss dehydration in older people [18]. In hyponatremia, a classic study cited in a recent systematic review showed that experienced physicians correctly classified only about 47% of hypovolemic and 48% of normovolemic patients on clinical grounds [58].

POCUS offers a rapid, repeatable and radiation-free complement to clinical examination. A 2026 systematic review identified 11 studies on POCUS in hyponatremia, mostly case reports, case series and small cohorts, the largest including 110 patients [58]. The main elements were the inferior vena cava (IVC), lung ultrasound and venous Doppler assessed with the venous excess ultrasound (VExUS) score. In a single small prospective study of 19 patients with sodium below 120 mmol/L, the agreement between the initial diagnosis and the final cause of hyponatremia increased from 63% with clinical assessment alone to 84% when POCUS was added [58]. Because of its size, this result is hypothesis-generating and cannot be generalized to all older adults with hyponatremia, nor to those with milder or drug-associated forms. Higher VExUS grades were associated with difficulty in correcting hyponatremia and with diuretic resistance [58]. The evidence is limited: no accuracy figures for distinguishing hypovolemia from euvolemia were available, the technique is operator-dependent, and a collapsible IVC may also be seen in SIAD [58].

For drug-induced hyponatremia, the potential value of POCUS, as an adjunct to clinical and laboratory assessment rather than a replacement for it, lies in helping to separate three common situations: sodium and volume loss with thiazides, SIAD with antidepressants, antiepileptic drugs or other SIAD-inducing drugs, and hypervolemic hyponatremia in patients with heart failure treated with loop diuretics. These situations require opposite treatments, from isotonic fluid to fluid restriction or decongestion. POCUS findings should be interpreted together with the medication history and urine indices, not in isolation, and its routine benefit in this setting has not been established. The main ultrasound findings and their interpretation are summarized in Table 6.

### 8.4. Severity-Based Management and Disposition

The medication-focused assessment described above should be integrated with severity-based management, so that patients who require immediate stabilization are distinguished from those who can be observed or discharged after medication adjustment. A practical approach is summarized in Table 7 and follows four steps.

First, severity is defined by symptoms and ECG findings, not by the laboratory value alone. Severe symptoms of hyponatremia (vomiting, seizures, reduced consciousness or cardiorespiratory distress), ECG changes or arrhythmias with dyskalemia, and tetany, seizures or QT prolongation with hypocalcemia or hypomagnesemia require immediate treatment and continuous monitoring, whatever the measured value [19,30,33,34].

Second, potentially spurious results should be confirmed when they do not fit the clinical picture, provided that this does not delay emergency treatment. Pseudohyperkalemia may result from fist clenching, prolonged tourniquet use, hemolysis or marked leukocytosis or thrombocytosis, and should be excluded by repeat measurement [33]. Hyponatremia should be confirmed as hypotonic by measuring serum osmolality, and hyperglycemia should be excluded; in severe hyperlipidemia or hyperproteinemia, indirect methods that dilute the sample may underestimate sodium, and direct measurement on an undiluted sample, for example on a blood gas analyzer, avoids this error [19,20]. Total calcium should be interpreted in relation to albumin or confirmed by ionized calcium.

Third, volume status and the offending drug determine the specific treatment. In hyponatremia without severe symptoms, isotonic saline is appropriate for diuretic-induced hypovolemia, fluid restriction for drug-induced SIAD and decongestion for hypervolemic states (Section 8.3) [19,20,58]. The offending drug should be stopped or held in severe disturbances and in most moderate ones, whereas in mild disturbances dose reduction and correction of contributing factors may be sufficient, particularly for drugs with proven benefit such as RAAS inhibitors [32,33,40].

Fourth, correction must be controlled and monitored serially. In chronic hyponatremia, which is the rule in older patients with drug-induced hyponatremia, the increase in serum sodium should not exceed 10 mmol/L in the first 24 h and 8 mmol/L in each subsequent 24 h, and lower targets are advised in patients at high risk of osmotic demyelination, such as those with a sodium concentration of 105 mmol/L or less, hypokalemia, alcohol use disorder, malnutrition or advanced liver disease [19,20]. Withdrawal of a thiazide, restoration of volume or correction of hypokalemia can trigger a brisk water diuresis. Sodium should therefore be measured repeatedly during active correction, for example after 1, 6 and 12 h, together with urine output, and if the limit is exceeded, sodium should be re-lowered with electrolyte-free water, with or without desmopressin [19,20]. After emergency treatment of hyperkalemia, potassium, the ECG and, after insulin, blood glucose should be reassessed frequently, because hypoglycemia is a potential complication [33]. In chronic hypernatremia, sodium should be lowered slowly, by less than 0.5 mmol/L per hour and less than 12 mmol/L per day [38].

Disposition depends on severity, the response to initial treatment and the need for serial monitoring. Admission to a monitored bed is indicated for patients who require hypertonic saline, emergency treatment of hyperkalemia, or intravenous potassium, magnesium or calcium with ECG monitoring, and for those with ECG changes, acute kidney injury or a need for frequent electrolyte measurements. Patients with moderate disturbances who respond to initial treatment can be managed in an observation unit until a repeat measurement confirms stability. Patients with mild, asymptomatic disturbances, stable kidney function, a probable drug contributor that has been identified and modified, and a reliable plan for laboratory follow-up can usually be discharged (Section 9.3). Because no validated disposition criteria exist for drug-associated electrolyte disturbances, these thresholds represent the authors’ proposal based on the evidence reviewed and should be adapted to local resources and to the patient’s frailty and social circumstances [5].

Figure 2 integrates these elements into a structured ED workflow. The first step is to assess severity: patients with severe symptoms, ECG changes or marked hyperkalemia require immediate stabilization (Table 7), doubtful results are confirmed without delaying treatment, and other causes of the presentation are considered in parallel (step 0). The medication history then identifies recently started or changed drugs, combinations, non-prescription products and past treatments (step 1); laboratory and ECG assessment is adapted to these drugs (step 2); and volume status, assessed clinically and, when uncertain, with POCUS as an adjunct (step 3), guides the treatment of hyponatremia, while each of the other disturbances has its own targeted actions. Finally, the discharge step organizes the transition of care in time: documentation and adjustment of drugs at discharge, communication with the prescriber, early laboratory follow-up and ongoing structured medication review (step 4; Section 9.2 and Section 9.3).

## 9. Management and Prevention

### 9.1. Management of the Offending Drug

In most cases, the offending drug should be stopped or held during the acute episode, and the decision to restart it requires a balance between its benefit and the risk of recurrence [6]. The cause of the episode, for example a recently started drug, a course of trimethoprim or an intercurrent dehydration, should be documented so that it can be avoided in the future [14]. The main decisions differ between drug classes.

Thiazides. Given the high absolute risk of profound hyponatremia in women aged 80 years or older, an alternative antihypertensive drug is usually preferable after an episode of profound thiazide-induced hyponatremia [23]. Calcium channel blockers carried a lower risk than thiazides in the same cohort [23], and ongoing treatment with other antihypertensive classes was not associated with severe hyponatremia [36]. After an episode of thiazide-associated hypercalcemia, PTH should be measured, because primary hyperparathyroidism is found in about one in four patients [24].

Antidepressants and antiepileptic drugs. For SSRIs, the risk is highest at initiation and declines with continued treatment, so a cautious rechallenge with close sodium monitoring, or a switch to an agent with lower risk such as mirtazapine, may be considered [6,25]. With oxcarbazepine and carbamazepine, the risk persists during treatment [6,13], and a change in antiepileptic drug should be discussed with the treating neurologist or psychiatrist rather than decided in the ED.

PPIs. An evidence-based guideline recommends deprescribing, by dose reduction, stopping or on-demand use, in adults who have completed at least four weeks of treatment for heartburn or mild to moderate reflux disease or esophagitis and whose symptoms have resolved; it does not apply to patients with Barrett esophagus, severe esophagitis or a history of bleeding ulcers [59]. After severe hypomagnesemia, observational data support stopping the PPI: recurrence was about half as frequent when the PPI was stopped [49].

RAAS inhibitors and MRAs. The aim is to treat the hyperkalemia and to maintain, rather than abandon, a drug with proven benefit in heart failure and chronic kidney disease [32,33]. Withdrawal at discharge is common and has been associated with worse survival [40]. During the transition of care, recurrence of hyperkalemia can be reduced by three complementary measures: (1) individualized dose adjustment with early, scheduled monitoring of potassium and renal function, for example before and 1–2 weeks after an ACE inhibitor or angiotensin II receptor blocker is restarted or its dose is changed [32]; (2) potassium binders, which in the DIAMOND trial reduced hyperkalemia and allowed MRA therapy to be maintained [45]; and (3) assessment of eligibility for SGLT2 inhibitors, which reduced the risk of serious hyperkalemia in people with type 2 diabetes [44]. These measures should be individualized to the patient’s comorbidities, and evidence in very old and frail patients is limited (Section 11). Precipitating drugs, such as trimethoprim and NSAIDs, should be avoided in these patients when alternatives exist [14,42].

Denosumab. The risk of severe hypocalcemia after denosumab was highest in patients with an eGFR below 15 mL/min/1.73 m^2^ or on dialysis, and only about one third of denosumab users have calcium measured in the following months [51]. After an ED presentation with hypocalcemia, the decision about the next dose should be made with the prescriber, and calcium monitoring should be planned.

### 9.2. Prevention and the Role of the Emergency Department

Several types of ED-based programs have been developed to improve medication safety in older adults. In addition to pharmacist review and computerized decision support (Section 8.1), clinician education was associated with a 19% reduction in inappropriate prescribing [57]. In a Veterans Affairs ED, a quality improvement initiative combining academic detailing, electronic alerts and individual feedback reduced inappropriate prescribing to older adults by 47.5% [60]. These programs now form part of the Geriatric Emergency Department Guidelines 2.0, the first emergency medicine subspecialty guidelines developed with the GRADE methodology [61].

The evidence for each component should be judged by the outcome it has been shown to change, and improvements in prescribing appropriateness should be distinguished from effects on clinical outcomes (Table 8).

Medication reconciliation. Discrepancies between recorded and actual medication use are frequent in the ED [56], and reconciliation is a prerequisite for recognizing a drug-associated disturbance. In a Cochrane review of 25 randomized trials including 6995 participants, reconciliation at transitions of care reduced medication discrepancies (risk ratio 0.53), but the certainty of this evidence was very low, and its effects on adverse drug events (risk ratio 1.09; low certainty) and unplanned readmissions (risk ratio 0.72; moderate certainty of little or no difference) remained uncertain [62].

Pharmacist-led interventions. In older ED patients, clinical pharmacist review increased the deprescribing of potentially inappropriate medications (odds ratio 0.68) but was not associated with fewer hospital admissions (odds ratio 0.86), a shorter length of stay or fewer ED revisits, and only one of the included studies was at low risk of bias [57]. Reviews of fall risk-increasing drugs did not reduce falls at 12 months [57]. Outside the ED, a randomized trial in 1467 medical inpatients taking five or more drugs (median age 72 years) tested a multicomponent intervention combining medication review, reconciliation at discharge, a motivational interview and follow-up with primary care. It reduced readmission within 30 days (14.3% vs. 22.3%; hazard ratio 0.62) and the composite of readmission or ED visit within 180 days (hazard ratio 0.77), but not drug-related readmissions or all-cause mortality [63]. Multicomponent programs that extend beyond the index visit therefore appear the most promising, but their effect on electrolyte-related events has not been reported.

Electronic prescribing alerts and decision support. Computerized decision support reduced the ordering of potentially inappropriate medications in older ED patients (odds ratio 0.60), and one study reported fewer adverse drug events (3.4% vs. 7.1%) [57]. No ED study has evaluated alerts targeted at drug combinations that cause electrolyte disturbances, such as trimethoprim or NSAIDs in patients taking RAAS inhibitors.

Post-discharge laboratory monitoring. Guidance recommends measuring potassium and creatinine before and 1–2 weeks after starting an ACE inhibitor or angiotensin II receptor blocker, and at defined intervals after starting an MRA [32], and the AGS Beers Criteria advise close sodium monitoring when SIAD-inducing drugs are started or their dose is changed [16]. These recommendations are based on expert opinion, adherence in routine practice is limited [33], and only about one third of patients starting denosumab have calcium measured [51]. Whether such monitoring prevents ED visits, admissions or arrhythmias has not been tested.

Communication with primary care. In the Spanish cohort of drug-induced hyponatremia, treatment changes at discharge were associated with ED revisits in univariable analysis [5], and in patients admitted with hyperkalemia, RAAS inhibitors were stopped at discharge in about one third and their withdrawal was associated with worse survival [40]. Structured communication of the adverse drug event and of the plan for each drug is therefore reasonable, and it was part of the only intervention shown to reduce readmissions [63], but its specific contribution has not been isolated.

In summary, ED-based interventions have been shown to improve prescribing appropriateness, and multicomponent pharmacist-led programs can reduce readmissions, but no intervention has been shown to reduce recurrent electrolyte disturbances, electrolyte-related admissions, arrhythmias or mortality, because none has specifically targeted these outcomes. Nevertheless, the ED visit offers a unique opportunity: the adverse drug event has just occurred, the causal drug can often be identified, and both the patient and the prescriber are likely to accept a change. Explicit criteria such as the Beers Criteria and STOPP/START provide a structured framework for this review [16,17].

### 9.3. Follow-Up After the Emergency Department Visit

Prevention also depends on what happens after the ED. Figure 2 (step 4) organizes the post-discharge actions in time: documentation of the adverse drug event and adjustment of the drugs involved at discharge, communication with the prescriber or primary care physician, early laboratory follow-up, and ongoing structured medication review. The AGS Beers Criteria recommend close monitoring of sodium when diuretics, antidepressants, carbamazepine, oxcarbazepine, antipsychotics or tramadol are started or their dose is changed, but do not specify the timing [16]. Based on the early peak of risk observed in population-based studies [22,23,25,27], we suggest that this check be performed within the first one to two weeks after a thiazide or an SSRI is started in older adults, particularly in women aged 80 years or older [23] and in patients taking other sodium-lowering drugs [13]. For PPIs, the early increase in relative risk is accompanied by a low absolute risk, and routine sodium monitoring is not recommended [27]. These suggestions represent the authors’ interpretation of observational data; the optimal monitoring schedule has not been established in trials. After an ED visit for a drug-associated disturbance, early laboratory follow-up appears reasonable given the frequency of revisits [5], although its effect on recurrence has not been studied. Communicating the adverse drug event to the primary care physician, and recording it as an intolerance where appropriate, may help prevent re-exposure. When a disturbance recurs, however, re-exposure to the drug or non-adherence to the agreed changes should not be assumed to be the only explanation. Progression of the underlying disease, such as heart failure or chronic kidney disease, changes in dietary or solute intake, such as a low-solute “tea and toast” diet [9], and occult infection or malignancy, which was the most common identifiable cause of severe hypercalcemia in the ED [50], should also be considered.

## 10. Causality, Confounding and Multiple Contributors

The strength of the causal link between a drug and an electrolyte disturbance varies considerably across the evidence summarized in Section 4, Section 5 and Section 6, and three levels can be distinguished. First, for a few drugs a causal effect on potassium has been demonstrated in randomized trials: in the FIDELITY pooled analysis, hyperkalemia occurred in 14.0% of patients treated with finerenone and 6.9% of those receiving placebo [41], and SGLT2 inhibitors reduced the risk of serious hyperkalemia in a meta-analysis of randomized trials [44]. Second, for several drugs a causal role is strongly supported by a direct pharmacological mechanism together with a steep, time-dependent pattern of risk in population-based studies: desmopressin acts directly on the renal vasopressin V2 receptor [31], trimethoprim blocks distal sodium channels [14], and the risk of hyponatremia with thiazides and SSRIs peaks in the first week of treatment and declines thereafter [22,25]. Third, for other associations, such as those of PPIs or antipsychotics with hyponatremia, the absolute risks are small and residual confounding cannot be excluded [26,27].

Observational estimates are subject to several sources of bias. Confounding by indication arises when the condition for which a drug is prescribed itself affects electrolyte balance, for example heart failure or chronic kidney disease in patients receiving RAAS inhibitors or MRAs [32,33], or the gastrointestinal symptoms for which PPIs are started [27]. Detection bias may arise because electrolytes are measured more often in sicker patients, whereas in other situations they are rarely measured at all; only about one third of patients starting denosumab had calcium checked in the following months [51]. In addition, registry-based studies capture drug dispensing rather than actual intake, and the medication lists available in the ED often differ from what patients really take [56].

In the individual older patient, a drug is rarely the only cause. Hyponatremia usually results from the interaction of a drug with age-related changes in water handling, low solute intake, intercurrent illness and other medications (Section 3.1) [9]. Hyperkalemia in patients receiving RAAS inhibitors occurs mainly in those with reduced GFR, heart failure or diabetes [32,33], and higher potassium concentrations were associated with chronic kidney disease and heart failure in a large hospital cohort [40]. Acute illness often acts as the trigger: NSAID-associated hyperkalemia is closely linked to acute kidney injury [42], and infections caused about half of the hypernatremia episodes in older adults in a Swedish cohort [37]. Concomitant medications frequently determine whether a risk becomes clinically relevant: the excess risk of hyperkalemia with trimethoprim appeared more pronounced in patients taking RAAS inhibitors or spironolactone [14], the association between PPIs and hypomagnesemia was confined to patients also taking diuretics [28], and combinations of psychotropic drugs with other hyponatremia-inducing agents increased the risk of hyponatremia many-fold [13]. Polypharmacy is therefore both a confounder in epidemiological studies and a true effect modifier at the bedside (Section 3.2).

For the emergency physician, causality is best judged at the level of the individual patient, using the elements that underlie formal causality assessment: the temporal relationship with drug initiation or dose change, a plausible mechanism, the exclusion of alternative causes and the response to withdrawal of the drug. A drug identified as a probable contributor should be documented as such, while the other contributing factors are treated in parallel.

## 11. Knowledge Gaps and Future Directions

Several important questions remain open. First, most data on drug-induced electrolyte disturbances come from population-based registries and pharmacovigilance databases, which identify hospitalized cases but rarely capture the ED visit itself [22,23,28]. Prospective ED studies that record medication exposure, timing of initiation, combinations and outcomes are scarce, and the most recent ED study of drug-induced hyponatremia was retrospective and conducted in a single center [5]. Data on drug-induced hypomagnesemia and hypocalcemia in unselected ED patients are almost absent, largely because magnesium and calcium are not measured routinely; even after denosumab is started, only about one third of patients have calcium checked [51].

Second, the optimal strategy for laboratory monitoring after starting high-risk drugs in older adults has not been tested in trials. The early peak of risk with thiazides, SSRIs and PPIs is well documented [22,25,27], and the higher absolute risk in older women is now clear [23,35], but whether a sodium check in the first weeks of treatment prevents ED visits is unknown. Similarly, it is not known whether ED-based medication review programs reduce recurrence of electrolyte disturbances, revisits or mortality, because none of the programs evaluated so far specifically targeted electrolyte disturbances [57].

Third, the role of POCUS in drug-induced hyponatremia rests on small studies without accuracy data for distinguishing hypovolemia from euvolemia [58]. Prospective studies in older ED patients, comparing a combined clinical, laboratory and ultrasound approach with usual care, are needed before it can be recommended routinely.

Finally, the balance between preventing electrolyte disturbances and maintaining drugs with proven benefit requires individualized decisions. RAAS inhibitors are frequently stopped after hyperkalemia, and this has been associated with worse survival [40], while evidence from the trials of potassium binders, SGLT2 inhibitors and finerenone in very old and frail patients is limited [41,44,45]. Future studies should evaluate interventions targeted specifically at drugs that affect electrolyte balance, such as automated alerts when an older patient on a RAAS inhibitor receives trimethoprim or an NSAID, when a thiazide is started in a woman aged 80 years or older, or when a patient with a history of lithium treatment presents with hypernatremia [14,23,37,42].

These gaps translate into five practical research priorities. First, prospective multicenter ED cohorts of older adults should record medication exposure, timing of initiation, combinations and outcomes, using standardized definitions of drug-associated electrolyte disturbances. Second, pragmatic trials should test early laboratory monitoring after high-risk drugs are started, such as sodium after a thiazide or SSRI and potassium after a RAAS inhibitor, with ED visits and hospital admissions as outcomes. Third, comparative cluster-randomized or pragmatic trials should evaluate, alone and in combination, three complementary preventive strategies: electronic health record-based prescribing alerts for high-risk combinations; post-discharge medication reconciliation by clinical pharmacists, including telehealth models; and early primary care follow-up with standardized electrolyte monitoring within seven days of ED discharge. These trials should use electrolyte-specific outcomes: recurrence of the disturbance, ED revisits, admissions, arrhythmias and mortality, in addition to prescribing appropriateness. Fourth, the diagnostic and prognostic value of POCUS and of severity-based disposition criteria, such as those proposed in Table 7, should be validated prospectively. Fifth, very old and frail patients should be included in trials of potassium binders, SGLT2 inhibitors and finerenone, so that drugs with proven benefit can be maintained safely in this population.

## 12. Conclusions

Drug-associated electrolyte disturbances are a common and potentially preventable reason for ED visits in older adults. Thiazide diuretics, antidepressants, antiepileptic drugs, antipsychotics, PPIs and desmopressin are the drugs most often associated with hyponatremia; RAAS inhibitors, MRAs, trimethoprim and NSAIDs predispose to hyperkalemia, particularly in patients with chronic kidney disease; diuretics and PPIs are associated with hypokalemia and hypomagnesemia; thiazides and supplements contribute to hypercalcemia; denosumab is associated with a risk of severe hypocalcemia in advanced kidney disease; and lithium remains a well-documented cause of hypernatremia, through nephrogenic diabetes insipidus that may persist after treatment is stopped. Most of this evidence is observational, and in the individual patient, a drug usually acts together with comorbidities, acute illness and other medications. In observational studies, the risk with thiazides, SSRIs and PPIs appeared highest shortly after initiation; for thiazides, it also increases with age, especially in women, and for most drugs it is magnified by combinations. For the emergency physician, the key steps are a careful medication history that includes recent changes, past treatments and non-prescription products; targeted laboratory and ECG assessment; volume assessment in which POCUS can complement, but not replace, clinical and laboratory evaluation; cautious correction after the suspected drug is withdrawn; and communication with the prescriber at discharge, so that drugs with proven benefit are adjusted rather than abandoned. ED-based medication safety programs can reduce inappropriate prescribing, but their effect on electrolyte-related outcomes still needs to be demonstrated in prospective studies. Future studies should evaluate multifaceted transition-of-care strategies that go beyond medication reduction alone, including automated prescribing alerts, post-discharge medication review by clinical pharmacists and early primary care follow-up with standardized electrolyte monitoring. They should also determine how best to balance the continuation of essential therapies with electrolyte safety, through proactive dose adjustment during intercurrent dehydration, potassium binders to support indicated RAAS inhibitor therapy and the targeted use of SGLT2 inhibitors, and whether these approaches reduce recurrent electrolyte disturbances in older adults.

## Figures and Tables

**Table 1:** Main drugs associated with electrolyte disturbances in older adults presenting to the emergency department.

Drug or Class	Disturbance	Main Mechanism	Highest-Risk Period or Group	Ref.
Thiazide and thiazide-like diuretics	Hyponatremia; hypokalemia; hypercalcemia	Impaired urinary dilution; renal sodium and potassium loss; reduced urinary calcium excretion	First weeks after initiation; risk increases with age and is highest in women aged ≥80 years; hypercalcemia unmasks primary hyperparathyroidism in about one in four patients	[4,22,23,24]
SSRIs and SNRIs	Hyponatremia	SIAD	First weeks after initiation or dose increase	[13,25]
Oxcarbazepine, carbamazepine	Hyponatremia	Increased collecting duct water permeability (V2 receptor–aquaporin-2 pathway); SIAD-like effect	Throughout treatment, increasing with duration; higher with oxcarbazepine; psychotropic combinations	[6,13]
Atypical antipsychotics	Hyponatremia	SIAD	First 30 days after initiation (period studied); small absolute increase (0.06%)	[26]
Tramadol	Hyponatremia	SIAD	Initiation or dose change	[16]
Proton pump inhibitors	Hyponatremia; hypomagnesemia	Not fully established; reduced intestinal magnesium absorption	First week after initiation (hyponatremia); long-term use, concomitant diuretics (hypomagnesemia)	[27,28,29,30]
Desmopressin	Hyponatremia	V2 receptor agonism (antidiuresis)	First 30 days after initiation; older adults (mean age 70 years) treated for nocturia	[31]
ACE inhibitors, ARBs, MRAs	Hyperkalemia	Reduced aldosterone effect on renal potassium secretion	Reduced GFR; heart failure; diabetes; advanced age; concomitant potassium-raising drugs	[32,33]
Trimethoprim	Hyperkalemia	Amiloride-like blockade of distal sodium channels	Concomitant RAAS inhibitors or spironolactone	[14]
Loop diuretics	Hypokalemia; hypomagnesemia	Renal potassium and magnesium loss	Combination with thiazides or PPIs; vomiting, diarrhea or reduced intake	[4,30,34]

The risk is not uniform across older adults: it depends on renal function, concomitant medications, age, sex and acute illness (see Section 6.3 and Section 10). ACE, angiotensin-converting enzyme; ARBs, angiotensin II receptor blockers; GFR, glomerular filtration rate; MRAs, mineralocorticoid receptor antagonists; PPIs, proton pump inhibitors; RAAS, renin–angiotensin–aldosterone system; SIAD, syndrome of inappropriate antidiuresis; SNRIs, serotonin–norepinephrine reuptake inhibitors; SSRIs, selective serotonin reuptake inhibitors.

**Table 2:** Drug-associated potassium disturbances in older adults: typical emergency department scenarios and practical actions.

Drug or Class	Typical ED Scenario	Action in the ED	After the Acute Episode	Ref.
ACE inhibitors, ARBs	Hyperkalemia with acute kidney injury, dehydration or progression of CKD	Treat hyperkalemia; hold or reduce the dose temporarily if severe	Reintroduce or re-titrate when potassium is controlled; avoid permanent withdrawal without a clear decision	[32,33,40]
MRAs (spironolactone, eplerenone, finerenone)	Hyperkalemia in heart failure or diabetic kidney disease	Hold temporarily; check other potassium-raising drugs	Consider a potassium binder to allow continuation; monitor potassium	[41,45]
Trimethoprim, co-trimoxazole	Hyperkalemia a few days after an antibiotic for urinary tract infection in a patient on RAAS inhibitors or spironolactone	Stop; choose an alternative antibiotic	Avoid the combination when possible	[14]
NSAIDs	Hyperkalemia and/or acute kidney injury after a short course	Stop the NSAID	Review analgesia; ask about over-the-counter use	[42]
Potassium supplements	Severe hyperkalemia	Stop	Reassess the indication	[43]
Thiazide and loop diuretics	Hypokalemia with vomiting, diarrhea or reduced intake; weakness; arrhythmia	Replace potassium; measure magnesium; record an ECG	Review the dose; supplementation may not prevent recurrence	[4,30,34,46]
β_2_-agonists, insulin	Transient hypokalemia due to intracellular shift	Monitor potassium	Review dosing	[34]
QT-prolonging drugs	Hypokalemia with QT prolongation	Correct potassium and magnesium; review all QT-prolonging drugs	Reassess the need for each drug	[30,47]

ACE, angiotensin-converting enzyme; ARBs, angiotensin II receptor blockers; CKD, chronic kidney disease; ECG, electrocardiogram; MRAs, mineralocorticoid receptor antagonists; NSAIDs, non-steroidal anti-inflammatory drugs; RAAS, renin–angiotensin–aldosterone system.

**Table 3:** Main drug classes associated with electrolyte disturbances in older adults: mechanisms, patient-level risk factors, typical presentation and suggested monitoring.

Drug or Class	Disturbance (Mechanism)	Patient-Level Risk Factors	Typical Presentation	Suggested Monitoring	Ref.
Thiazide and thiazide-like diuretics	Hyponatremia (impaired urinary dilution); hypokalemia and hypomagnesemia (renal loss); hypercalcemia (reduced urinary calcium excretion)	Age ≥ 80 years; female sex; first weeks of treatment; low solute intake; concomitant SIAD-inducing drugs; calcium or vitamin D supplements; primary hyperparathyroidism	Confusion, nausea, vomiting, falls; muscle weakness; polyuria and constipation (hypercalcemia)	Sodium and potassium after initiation or dose change ^a^; calcium and PTH if hypercalcemia ^b^	[16,22,23,24,35]
Loop diuretics	Hypokalemia and hypomagnesemia (NKCC2 inhibition, renal loss); hyponatremia	Combination with thiazides or PPIs; vomiting or diarrhea; heart failure; female sex (hyponatremia)	Weakness; arrhythmias; QT prolongation	Potassium, magnesium and creatinine; ECG if QT-prolonging drugs are taken ^b^	[4,28,30,34,35,47]
SSRIs, SNRIs	Hyponatremia (SIAD)	First weeks after initiation or dose increase; women aged ≥ 65 years; concomitant diuretics or other SIAD-inducing drugs	Confusion, gait instability, falls	Sodium at initiation or dose change ^a^	[13,16,25]
Oxcarbazepine, carbamazepine	Hyponatremia (increased renal sensitivity to vasopressin)	Oxcarbazepine > carbamazepine; longer treatment; combination with other psychotropic drugs	Confusion, gait instability, falls	Sodium at initiation or dose change ^a^ and during long-term treatment ^b^	[6,13,16]
Atypical antipsychotics	Hyponatremia (SIAD)	First 30 days after initiation	Confusion, often masked by delirium or dementia	Sodium at initiation or dose change ^a^	[16,26]
Tramadol	Hyponatremia (SIAD)	Combination with antidepressants or diuretics	Confusion, falls	Sodium at initiation or dose change ^a^	[16]
Desmopressin	Hyponatremia (vasopressin V2 receptor agonism)	Older age; early treatment	Confusion, headache, seizures	Sodium early after initiation ^b^	[31]
Proton pump inhibitors	Hypomagnesemia (reduced intestinal absorption), with secondary hypokalemia and hypocalcemia; hyponatremia (mechanism not established)	Long-term and high-dose use; concomitant diuretics; female sex; diabetes; low body mass index; renal impairment	Refractory hypokalemia or hypocalcemia; tetany; arrhythmias; QT prolongation	Magnesium in long-term users taking diuretics or QT-prolonging drugs ^b^	[27,28,29,30,49]
Lithium (current or past)	Hypernatremia (nephrogenic diabetes insipidus; reduced aquaporin-2 expression)	Treatment ≥ 5 years; impaired thirst; restricted access to water; intercurrent infection	Polyuria, thirst, confusion	Sodium; ask about current and past use ^b^	[37,38]
ACE inhibitors, ARBs, MRAs (including finerenone)	Hyperkalemia (reduced aldosterone effect on renal potassium secretion)	Reduced GFR; heart failure; diabetes; potassium supplements; concomitant trimethoprim or NSAIDs; dehydration	Weakness; bradycardia; conduction disturbances	Potassium and creatinine before and 1–2 weeks after starting an ACE inhibitor or ARB, at 1 and 2 weeks after starting an MRA, after dose changes and during intercurrent illness ^c^	[32,33,40,41]
Trimethoprim, co-trimoxazole	Hyperkalemia (amiloride-like blockade of distal sodium channels)	Concomitant RAAS inhibitors or spironolactone; reduced creatinine clearance	Hyperkalemia a few days after an antibiotic course	Avoid the combination when possible ^a^; otherwise check potassium ^b^	[14,16]
NSAIDs	Hyperkalemia with acute kidney injury (impaired renin release)	Concomitant RAAS inhibitors or diuretics; chronic kidney disease; dehydration	Rising creatinine; hyperkalemia after a short course	Creatinine and potassium in high-risk patients ^b^	[33,42]
Denosumab	Hypocalcemia (inhibition of osteoclast-mediated bone resorption)	eGFR < 30 mL/min/1.73 m^2^; dialysis	Paresthesia, tetany, seizures, QT prolongation	Calcium before and after injection in advanced chronic kidney disease ^b^	[51]
Calcium and vitamin D supplements	Hypercalcemia (increased calcium load)	Concomitant thiazides; over-the-counter use	Confusion, polyuria, constipation	Calcium; ask specifically about over-the-counter use ^b^	[24,50]

^a^ Recommended by the 2023 American Geriatrics Society Beers Criteria [16]. ^b^ Suggestion of the authors based on observational data; the optimal monitoring schedule has not been tested in trials. ^c^ Recommended in clinical guidance on hyperkalemia [32,33]. ACE, angiotensin-converting enzyme; ARBs, angiotensin II receptor blockers; ECG, electrocardiogram; eGFR, estimated glomerular filtration rate; GFR, glomerular filtration rate; MRAs, mineralocorticoid receptor antagonists; NKCC2, sodium–potassium–2-chloride cotransporter; NSAIDs, non-steroidal anti-inflammatory drugs; PPIs, proton pump inhibitors; PTH, parathyroid hormone; RAAS, renin–angiotensin–aldosterone system; SIAD, syndrome of inappropriate antidiuresis; SNRIs, serotonin–norepinephrine reuptake inhibitors; SSRIs, selective serotonin reuptake inhibitors.

**Table 4:** Key studies on drug-induced electrolyte disturbances relevant to older adults in emergency and hospital settings.

Study	Population and Design	n	Main Findings	Ref.
Arampatzis et al., 2013	ED patients (mean age 52 years), single-center cross-sectional	22,239	Diuretic users vs. non-users: hyponatremia 20% vs. 7.7%; hypokalemia 17% vs. 11%; hyperkalemia 13% vs. 4%	[4]
Mannheimer et al., 2021	Population-based case–control, Sweden	Nationwide registers	Thiazides: about 27% of hyponatremia hospitalizations; aOR 48 in first week vs. 2.9 in long-term use	[22]
Bergh Fahlén et al., 2026	New thiazide vs. calcium channel blocker users, cohort	159,080	Two-year incidence of Na^+^ < 125 mmol/L: 2.40% at ≥80 years; 3.06% in women ≥ 80 years (NNH 53)	[23]
Mannheimer et al., 2021	Population-based case–control, Sweden	Nationwide registers	SSRIs: aOR 29 for hyponatremia hospitalization in week 1, 2.1 by week 13	[25]
Gandhi et al., 2016	Adults ≥65 years starting atypical antipsychotics, matched cohort	58,008 per group	Hyponatremia hospitalization within 30 days 0.15% vs. 0.09% (RR 1.62)	[26]
Issa et al., 2023	Population-based case–control, Sweden; mean age 76 years	11,213 cases	Omeprazole/esomeprazole: aOR 6.87 in first week; 1.10 with ongoing use	[27]
Fralick et al., 2019	New desmopressin vs. oxybutynin users, cohort	3137 per group	Hyponatremia 146 vs. 11 per 1000 person-years (HR 13.2); mean age 70 years	[31]
Crellin et al., 2018	Adults ≥ 65 years with UTI, cohort	≈1.19 million	Trimethoprim vs. amoxicillin: hyperkalemia OR 2.27; AKI OR 1.72; about 18 extra hyperkalemia cases per 1000 with RAAS inhibitors or spironolactone	[14]
Nash et al., 2019	Adults ≥ 66 years starting NSAIDs, matched cohort	46,107 per group	Hyperkalemia within 30 days 0.40% vs. 0.27% (OR 1.50); AKI 0.82% vs. 0.59% (OR 1.41)	[42]
Zipursky et al., 2014	Adults ≥66 years, case–control	366 cases	PPIs: hypomagnesemia hospitalization aOR 1.43; 1.73 with diuretics	[28]
Seah et al., 2023	Severe hypomagnesemia with PPI use, case–control; mean age 71.8 years	189 cases	Diuretics aOR 1.68; high-dose PPI aOR 1.96; recurrence 69.7% if PPI continued vs. 35.7% if stopped	[49]
Cowan et al., 2023	Adults > 65 years starting denosumab, cohort	59,151	Hypocalcemia within 180 days 0.6% overall; 24% with eGFR < 15 mL/min/1.73 m^2^ or dialysis	[51]
Grasa et al., 2026	ED patients with drug-induced hyponatremia, retrospective	141	Mean age 80.5 years; hydrochlorothiazide in 50.3% of implicated exposures; 11% revisited within 30 days	[5]

AKI, acute kidney injury; aOR, adjusted odds ratio; ED, emergency department; eGFR, estimated glomerular filtration rate; HR, hazard ratio; NNH, number needed to harm; NSAIDs, non-steroidal anti-inflammatory drugs; OR, odds ratio; PPI(s), proton pump inhibitor(s); RAAS, renin–angiotensin–aldosterone system; RR, relative risk; SSRIs, selective serotonin reuptake inhibitors; UTI, urinary tract infection.

**Table 5:** Medication-focused checklist for older adults presenting to the emergency department with an electrolyte disturbance.

Step	Action in the ED	Rationale	Ref.
0. Severity	Assess symptoms and ECG; stabilize immediately if severe (Section 8.4); consider other causes of the presentation in parallel	Severity, not the laboratory value alone, determines urgency; electrolyte disturbances may not fully explain the presentation	[19,33]
1. Medication history	Complete list, including over-the-counter drugs, supplements and injectables; confirm with family, pharmacy or records	Discrepancies between recorded and actual medication use are frequent	[56]
2. Timing	Ask about drugs started or changed in the previous weeks	In observational studies, the risk with thiazides, SSRIs, PPIs and desmopressin was highest early after initiation	[22,23,25,27,31]
3. Combinations	Look for SSRI or antiepileptic plus diuretic; trimethoprim plus RAAS inhibitor or MRA; PPI plus diuretic	Combined exposure is associated with a higher risk	[13,14,28,49]
4. Laboratory	Sodium, potassium, creatinine; magnesium if diuretics or PPIs; calcium, phosphate and magnesium in hypocalcemia, particularly after denosumab in advanced CKD; serum osmolality, urine sodium and osmolality in hyponatremia	Hypomagnesemia is often unmeasured; urine indices help separate diuretic effect from SIAD	[19,30,51]
5. ECG	Record in dyskalemia and with QT-prolonging drugs	Electrolyte disturbances are associated with a higher risk of drug-induced arrhythmias	[47]
6. Volume status	Combine clinical examination with POCUS as an adjunct when volume status is uncertain	Clinical signs of volume depletion are unreliable in older adults	[18,58]
7. Treatment	Stop or hold the suspected drug; treat according to severity (Section 8.4); monitor sodium for rapid rise after withdrawal	Risk of overcorrection after removal of the cause	[19,20]
8. Discharge	Document the adverse drug event; communicate with the prescriber; plan early laboratory follow-up; involve a clinical pharmacist where available	Revisits are frequent; the ED visit is an opportunity for deprescribing	[5,57]

CKD, chronic kidney disease; ECG, electrocardiogram; ED, emergency department; MRA, mineralocorticoid receptor antagonist; PPI(s), proton pump inhibitor(s); RAAS, renin–angiotensin–aldosterone system; SIAD, syndrome of inappropriate antidiuresis; SSRI(s), selective serotonin reuptake inhibitor(s).

**Table 6:** Point-of-care ultrasound findings in older adults with hyponatremia and their interpretation in the context of drug exposure.

Ultrasound Finding	Suggested Volume State	Possible Drug-Related Context	Limitations	Ref.
Small IVC with >50% inspiratory collapse	Hypovolemia	Consistent with thiazide- or loop-diuretic-induced volume contraction, if supported by the history and urine indices	May also be seen in SIAD; depends on respiratory effort	[58]
Plethoric IVC with minimal collapse	Hypervolemia or raised right atrial pressure	May be associated with heart failure or advanced CKD treated with loop diuretics	May be distended in conditions unrelated to volume status	[58]
Predominant A-lines on lung ultrasound	Hypovolemia or euvolemia	Compatible with diuretic-associated hypovolemia or drug-associated SIAD	Does not distinguish between the two	[58]
Diffuse bilateral B-lines	Interstitial congestion	Suggests hypervolemic hyponatremia; isotonic fluid is not appropriate	Operator-dependent; limited data in hyponatremia	[58]
VExUS grade II–III	Significant venous congestion	Suggests congestion in patients on loop diuretics; associated with difficult correction and diuretic resistance	Small studies; moderate correlation with right atrial pressure	[58]

CKD, chronic kidney disease; IVC, inferior vena cava; SIAD, syndrome of inappropriate antidiuresis; VExUS, venous excess ultrasound.

**Table 7:** Severity-based emergency department management and disposition of drug-associated electrolyte disturbances in older adults.

Disturbance	Immediate Stabilization (Monitored Bed)	Observation and Targeted Treatment	Drug Adjustment and Possible Discharge	Serial Monitoring and Safety Limits	Ref.
Hyponatremia	Severe symptoms (vomiting, seizures, reduced consciousness, cardiorespiratory distress), whatever the sodium value: 150 mL 3% NaCl over 20 min, with sodium checked after each bolus and repeated up to two further times until sodium has risen by 5 mmol/L; stop all sodium-lowering drugs	Moderate symptoms (nausea, confusion, headache): consider a single 150 mL bolus of 3% NaCl over 20 min. Sodium < 125 mmol/L or a fall in a frail patient: confirm hypotonicity; stop the offending drug; treat according to volume status (0.9% saline or balanced crystalloid at 0.5–1.0 mL/kg/h if diuretic-induced hypovolemia, fluid restriction as first-line treatment if SIAD, decongestion if hypervolemic)	Sodium 130–135 mmol/L, asymptomatic, cause identified: stop or replace the drug; recheck sodium within a few days ^a^	Increase ≤10 mmol/L in the first 24 h and ≤8 mmol/L in each subsequent 24 h; lower targets if high risk of osmotic demyelination (sodium ≤ 105 mmol/L, hypokalemia, alcohol use disorder, malnutrition, advanced liver disease); sodium after 1, 6 and 12 h, then according to the response, and urine output; if the limit is exceeded, re-lower sodium with electrolyte-free water (oral water or 5% dextrose), with or without desmopressin	[19,20,58]
Hypernatremia	Reduced consciousness, seizures or shock: restore circulating volume with isotonic fluid first	Most older patients with sodium ≥ 150 mmol/L: isotonic saline first if severely hypovolemic, then hypotonic fluids; treat infection; withhold lithium, diuretics and osmotic laxatives	Rarely appropriate; mild hypernatremia only if oral intake is reliable and the cause is corrected ^a^	In chronic hypernatremia (>48 h or unknown duration), lower sodium by <0.5 mmol/L/h and <12 mmol/L per day; repeated sodium measurements; ask about past lithium use	[10,37,38]
Hyperkalemia	Potassium > 6.0 mmol/L or new ECG changes: cardiac monitoring; IV calcium; IV insulin with glucose; nebulized β_2_-agonist; stop all potassium-raising drugs; consider dialysis if potassium remains >6.0 mmol/L or ECG changes do not respond to medical treatment	Potassium 5.5–6.0 mmol/L with acute kidney injury, rising creatinine or an ongoing cause: ECG; repeat sample; potassium binder; hold RAAS inhibitor or MRA temporarily; treat acute kidney injury and dehydration	Potassium 5.5–6.0 mmol/L, no ECG changes, stable creatinine: exclude pseudohyperkalemia; stop trimethoprim, NSAIDs or potassium supplements; review diet; maintain RAAS inhibitors where possible (potassium binders; dose reduction only if other measures fail); recheck potassium	Frequent reassessment of potassium, ECG and glucose after insulin (risk of hypoglycemia); repeat the measurement to exclude pseudohyperkalemia (fist clenching, prolonged tourniquet, hemolysis, marked leukocytosis or thrombocytosis), but do not delay treatment if ECG changes are present	[32,33]
Hypokalemia	Potassium < 2.5 mmol/L or cardiac complications: IV potassium chloride with continuous ECG monitoring; correct magnesium	Potassium 2.5–2.9 mmol/L, concomitant digoxin or QT-prolonging drugs, or vomiting preventing oral intake: oral or IV replacement; ECG; measure and correct magnesium	Potassium 3.0–3.4 mmol/L, asymptomatic: oral replacement; review diuretic dose and combinations	ECG in all patients; IV potassium usually 10 mmol/h, maximum 20 mmol/h; remeasure potassium after replacement; refractory hypokalemia suggests hypomagnesemia	[33,34,47,48]
Hypomagnesemia	Seizures or severe arrhythmias (including torsades de pointes): IV loading dose of magnesium sulfate (4–8 mmol over 5–10 min), then 25 mmol/day, with ECG monitoring	Severe (<0.4 mmol/L) or symptomatic hypomagnesemia, or hypomagnesemia with refractory hypokalemia or hypocalcemia: 25 mmol magnesium sulfate IV over 12–24 h	Mild, asymptomatic: oral magnesium salts (15–20 mmol/day); reassess the PPI and diuretic	In chronic kidney disease, reduce the dose by 50% and monitor magnesium more frequently (risk of hypermagnesemia); recheck potassium and calcium	[30,49]
Hypocalcemia	Tetany, seizures, laryngospasm, arrhythmia or QT prolongation: IV calcium gluconate with ECG monitoring; correct magnesium	Corrected calcium < 1.80 mmol/L, particularly after denosumab in advanced kidney disease: IV then oral calcium	Mild, asymptomatic: oral calcium and vitamin D; inform the prescriber before the next denosumab dose	Confirm with ionized calcium when albumin is abnormal; serial calcium until stable; check magnesium	[30,51]
Hypercalcemia	Marked neuropsychiatric symptoms, arrhythmia or acute kidney injury: IV fluids; stop thiazides and calcium/vitamin D supplements; bisphosphonate, calcitonin or loop diuretic in selected patients	Calcium ≥ 2.80 mmol/L: IV fluids; treat dehydration; search for malignancy and primary hyperparathyroidism	Mild, asymptomatic, drug or supplement cause: stop the drug; arrange PTH measurement	Serial calcium and creatinine; fluid balance; PTH after the acute episode	[24,50]

Severity categories, correction limits and treatment doses follow the cited guidelines and reviews [19,20,30,32,33,34,38]; IV calcium for symptomatic hypocalcemia is listed as standard emergency treatment. ^a^ thresholds for discharge and follow-up are proposed by the authors, because no validated disposition criteria exist for drug-associated electrolyte disturbances, and should be adapted to local resources and to the patient’s frailty and social circumstances. Admission to a monitored bed is indicated for all patients in the first column and for patients in the second column who do not respond to initial treatment or need frequent measurements. ECG, electrocardiogram; IV, intravenous; MRA, mineralocorticoid receptor antagonist; NSAIDs, non-steroidal anti-inflammatory drugs; PPI, proton pump inhibitor; PTH, parathyroid hormone; RAAS, renin–angiotensin–aldosterone system; SIAD, syndrome of inappropriate antidiuresis.

**Table 8:** Evidence for prevention and follow-up strategies: effects on prescribing versus clinical and electrolyte-related outcomes.

Strategy	Effect on Prescribing or Process	Effect on Clinical Outcomes	Electrolyte-Specific Evidence	Type of Evidence	Ref.
Medication reconciliation	Fewer medication discrepancies (RR 0.53; very low certainty)	No clear effect on adverse drug events (RR 1.09) or unplanned readmissions (RR 0.72)	None	Systematic review of 25 RCTs	[56,62]
Pharmacist review in the ED	More deprescribing of PIMs (OR 0.68)	No reduction in hospital admissions (OR 0.86), length of stay or ED revisits	None	Meta-analysis; only one study at low risk of bias	[57]
Multicomponent pharmacist intervention (review, reconciliation, patient interview, primary care follow-up)	Structured medication changes communicated to primary care	Fewer 30-day readmissions (HR 0.62) and fewer readmissions or ED visits at 180 days (HR 0.77); no effect on drug-related readmissions or mortality	Not reported	RCT in medical inpatients	[63]
Computerized decision support and electronic alerts	Less PIM ordering (OR 0.60); 47.5% less inappropriate prescribing in a multicomponent quality improvement program	Fewer adverse drug events in one study	No evaluation of alerts for electrolyte-related combinations	Meta-analysis; quality improvement study	[57,60]
Clinician education	Less PIM prescribing (OR 0.81)	Not demonstrated	None	Meta-analysis	[57]
Laboratory monitoring after starting high-risk drugs	Recommended for RAAS inhibitors, MRAs and SIAD-inducing drugs; adherence in practice is limited	Not tested	Timing based on the early peak of risk in observational studies	Guidance based on expert opinion; authors’ interpretation for timing	[16,22,23,25,27,32,33,51]
Early laboratory follow-up after an ED visit	—	Not tested	About 11% of patients with drug-induced hyponatremia return within 30 days	Authors’ suggestion based on observational data	[5]
Communication with the prescriber and documentation of the adverse drug event	Component of the multicomponent intervention above	Contribution not isolated	RAAS inhibitor withdrawal after hyperkalemia associated with worse survival	Observational data; reasonable but untested	[40,63]

ED, emergency department; HR, hazard ratio; MRAs, mineralocorticoid receptor antagonists; OR, odds ratio; PIMs, potentially inappropriate medications; RAAS, renin–angiotensin–aldosterone system; RCT, randomized controlled trial; RR, risk ratio; SIAD, syndrome of inappropriate antidiuresis.

## Data Availability

No new data were created or analyzed in this study. Data sharing is not applicable to this article.

## Supplementary Materials

The following supporting information can be downloaded at https://www.mdpi.com/article/10.3390/jcm15218719/s1. Table S1: PubMed/MEDLINE search strategy.

## Abbreviations

The following abbreviations are used in this manuscript:

ACE	angiotensin-converting enzyme
AGS	American Geriatrics Society
AKI	acute kidney injury
aOR	adjusted odds ratio
ARBs	angiotensin II receptor blockers
CKD	chronic kidney disease
ECG	electrocardiogram
ED	emergency department
EDEN	Emergency Department and Elder Needs
eGFR	estimated glomerular filtration rate
GFR	glomerular filtration rate
GRADE	Grading of Recommendations, Assessment, Development and Evaluation
HR	hazard ratio
ICU	intensive care unit
IV	intravenous
IVC	inferior vena cava
MRAs	mineralocorticoid receptor antagonists
NCC	sodium–chloride cotransporter
NKCC2	sodium–potassium–2-chloride cotransporter
NNH	number needed to harm
NSAIDs	non-steroidal anti-inflammatory drugs
OR	odds ratio
POCUS	point-of-care ultrasound
PPIs	proton pump inhibitors
PTH	parathyroid hormone
RAAS	renin–angiotensin–aldosterone system
RANKL	receptor activator of nuclear factor κB ligand
RR	relative risk
SGLT2	sodium–glucose cotransporter 2
SIAD	syndrome of inappropriate antidiuresis
SNRIs	serotonin–norepinephrine reuptake inhibitors
SSRIs	selective serotonin reuptake inhibitors
STOPP/START	Screening Tool of Older Persons’ Prescriptions/Screening Tool to Alert to Right Treatment
UTI	urinary tract infection
VExUS	venous excess ultrasound
