# Supplementary material for: Drug-Induced Electrolyte Disturbances in Older Adults Presenting to the Emergency Department: Mechanisms, Recognition and Prevention
Source: J Clin Med. 2026 Nov 8;15(21):8719. doi: 10.3390/jcm15218719 (PMC13764195; doi:10.3390/jcm15218719)
Supplement: Supplementary file 1 [file jcm-15-08719-s001.zip › jcm-4665023-supplementary.pdf]

Supplementary Materials

Drug-Induced Electrolyte Disturbances in Older Adults Presenting to the Emergency Department: Mechanisms, Recognition and Prevention

Table S1. PubMed/MEDLINE search strategy.

**Database**	PubMed/MEDLINE (https://pubmed.ncbi.nlm.nih.gov)
**Search date**	27 October 2026 (initial search on 3 October 2026)
**Publication period**	1 January 2016 – 30 September 2026
**Language**	English
**Concept 1: electrolyte disorders**	Hyponatremia, hypernatremia, hyperkalemia, hypokalemia, hypomagnesemia, hypocalcemia, hypercalcemia (MeSH and free-text terms)
**Concept 2: drug exposure**	Adverse drug events, drug-induced/drug-associated, diuretics, thiazides, antidepressants, antiepileptic drugs, antipsychotics, proton pump inhibitors, ACE inhibitors, angiotensin receptor blockers, mineralocorticoid receptor antagonists, NSAIDs, trimethoprim, desmopressin, lithium, denosumab
**Concept 3: population/setting**	Older adults, elderly, emergency department, hospitalization
**Full search string**	(("Hyponatremia"[MeSH] OR "Hypernatremia"[MeSH] OR "Hyperkalemia"[MeSH] OR "Hypokalemia"[MeSH] OR "Hypocalcemia"[MeSH] OR "Hypercalcemia"[MeSH] OR hyponatr*[tiab] OR hypernatr*[tiab] OR hyperkal*[tiab] OR hypokal*[tiab] OR hypomagnes*[tiab] OR hypocalc*[tiab] OR hypercalc*[tiab] OR dysnatr*[tiab] OR dyskal*[tiab]) AND ("Drug-Related Side Effects and Adverse Reactions"[MeSH] OR "adverse drug event*"[tiab] OR "drug-induced"[tiab] OR "drug-associated"[tiab] OR "Diuretics"[MeSH] OR diuretic*[tiab] OR thiazide*[tiab] OR "Antidepressive Agents"[MeSH] OR antidepressant*[tiab] OR "Anticonvulsants"[MeSH] OR antiepileptic*[tiab] OR "Antipsychotic Agents"[MeSH] OR antipsychotic*[tiab] OR "Proton Pump Inhibitors"[MeSH] OR "proton pump inhibitor*"[tiab] OR "Angiotensin-Converting Enzyme Inhibitors"[MeSH] OR "Angiotensin Receptor Antagonists"[MeSH] OR "Mineralocorticoid Receptor Antagonists"[MeSH] OR "Anti-Inflammatory Agents, Non-Steroidal"[MeSH] OR trimethoprim[tiab] OR desmopressin[tiab] OR lithium[tiab] OR denosumab[tiab]) AND ("Aged"[MeSH] OR elderly[tiab] OR "older adult*"[tiab] OR "older patient*"[tiab] OR "older people"[tiab] OR "Emergency Service, Hospital"[MeSH] OR "emergency department*"[tiab] OR hospitali*[tiab])) AND english[la] AND ("2016/01/01"[dp] : "2026/09/30"[dp])
**Records identified**	1742
**Additional sources**	Manual screening of reference lists of included articles and recent reviews
**References cited in the review**	63

ACE, angiotensin-converting enzyme; [dp], date of publication; [la], language; [MeSH], Medical Subject Headings; NSAIDs, non-steroidal anti-inflammatory drugs; [tiab], title/abstract. The search string can be pasted directly into the PubMed search box to reproduce the search.
